# A functional single-cell metabolic survey identifies *Elovl1* as a target to enhance CD8^+^ T cell fitness in solid tumours

**DOI:** 10.1038/s42255-025-01233-w

**Published:** 2025-03-10

**Authors:** Samantha Pretto, Qian Yu, Pierre Bourdely, Sarah Trusso Cafarello, Heleen H. Van Acker, Joren Verelst, Elena Richiardone, Lotte Vanheer, Amir Roshanzadeh, Franziska Schneppenheim, Charlotte Cresens, Maria Livia Sassano, Jonas Dehairs, Martin Carion, Shehab Ismail, Patrizia Agostinis, Susana Rocha, Tobias Bald, Johan Swinnen, Cyril Corbet, Sophia Y. Lunt, Bernard Thienpont, Mario Di Matteo, Massimiliano Mazzone

**Affiliations:** 1 Laboratory of Tumor Inflammation and Angiogenesis, Center for Cancer Biology, VIB, Leuven, Belgium; 2 Laboratory of Tumor Inflammation and Angiogenesis, Center for Cancer Biology, Department of Oncology, KU Leuven, Leuven, Belgium; 3 Laboratory for Functional Epigenetics, Department of Human Genetics, KU Leuven, Leuven, Belgium; 4 Pole of Pharmacology and Therapeutics (FATH), Institut de Recherche Expérimentale et Clinique (IREC), UCLouvain, Brussels, Belgium; 5 Department of Biochemistry and Molecular Biology, Michigan State University, East Lansing, MI USA; 6 Institute of Experimental Oncology (IEO), University Hospital Bonn, University of Bonn, Bonn, Germany; 7 Molecular Imaging and Photonics Division, Chemistry Department, Faculty of Sciences, KU Leuven, Heverlee, Belgium; 8 VIB BioImaging Core, Leuven, Belgium; 9 VIB-KU Leuven Center for Brain & Disease Research, Leuven, Belgium; 10 Cell Death Research and Therapy Group, Department of Cellular and Molecular Medicine, KU Leuven, Leuven, Belgium; 11 VIB Center for Cancer Biology, Leuven, Belgium; 12 Laboratory of Lipid Metabolism and Cancer, Department of Oncology, KU Leuven, Leuven, Belgium; 13 Department of Chemistry, KU Leuven, Heverlee, Belgium; 14 Department of Chemical Engineering and Materials Science, Michigan State University, East Lansing, MI USA

**Keywords:** Cancer immunotherapy, CD8-positive T cells, Metabolism, Pancreatic cancer, Immunology

## Abstract

Reprogramming T cell metabolism can improve intratumoural fitness. By performing a CRISPR/Cas9 metabolic survey in CD8^+^ T cells, we identified 83 targets and we applied single-cell RNA sequencing to disclose transcriptome changes associated with each metabolic perturbation in the context of pancreatic cancer. This revealed elongation of very long-chain fatty acids protein 1 (*Elovl1*) as a metabolic target to sustain effector functions and memory phenotypes in CD8^+^ T cells. Accordingly, *Elovl1* inactivation in adoptively transferred T cells combined with anti-PD-1 showed therapeutic efficacy in resistant pancreatic and melanoma tumours. The accumulation of saturated long-chain fatty acids in *Elovl1*-deficient T cells destabilized INSIG1, leading to SREBP2 activation, increased plasma membrane cholesterol and stronger T cell receptor signalling. *Elovl1*-deficient T cells increased mitochondrial fitness and fatty acid oxidation, thus withstanding the metabolic stress imposed by the tumour microenvironment. Finally, *ELOVL1* in CD8^+^ T cells correlated with anti-PD-1 response in patients with melanoma. Altogether, *Elovl1* targeting synergizes with anti-PD-1 to promote effective T cell responses.

## Abstract (web summary)

Through a functional CRISPR/Cas9 screening, Pretto et al. identify *Elovl1* as a target to improve antitumour immunity by remodelling T cell lipid metabolism.

## Main

Immunotherapies have revolutionized cancer treatment in the past decades. Immune-checkpoint blockade (ICB) drugs such as antibodies against programmed cell death 1 (PD-1), programmed death ligand 1 (PD-L1) or cytotoxic T-lymphocyte-associated antigen 4 (CTLA-4) (respectively, anti-PD-1, anti-PD-L1 and anti-CTLA-4) aim to reinvigorate tumour-infiltrating CD8^+^ T cells. These drugs have achieved high response rates of prolonged duration in subsets of patients with melanoma, renal cancer and lung cancer, but hardly show clinical benefit in immunologically cold tumours such as pancreatic ductal adenocarcinoma (PDAC)^1,2^. Similarly, adoptive T cell transfer (ACT) and chimeric antigen receptor (CAR) T cell approaches, which had striking success in blood cancers, showed disappointing results in treating solid tumours in general and PDAC in particular^3–5^. This underlines an urgent need for new strategies and adjuvant therapies to improve responses to currently available immunotherapies, as illustrated by the recent efforts to reinvigorate cytotoxic T cell responses through RNA-based neoantigen vaccination of patients with PDAC^6^ or the generation of antigen-specific CAR T cell therapy in murine PDAC models^7–9^. Cancer immunotherapies often lack efficacy because the microenvironment of solid tumours is hostile, with nutrient limitation, lactate-mediated acidification and hypoxia converging to suppress the infiltration and antitumoural activity of CD8^+^ T cells^2,10–13^. As the activity and differentiation of CD8^+^ T cells are regulated by different metabolic programmes, previous studies have focused on cancer and T cell metabolism to uncover vulnerabilities that can be exploited as new therapeutic options^14,15^. Metabolic genes regulating T cell differentiation, such as the mitochondrial pyruvate carrier (*Mpc*) and driving T cell exhaustion like protein-O-fucosyltransferase-1 (*Pofut1*), have been targeted to increase CD8^+^ T cell fitness in different tumour microenvironments (TMEs)^16,17^. Vice versa, treatment with the immune-checkpoint inhibitor anti-PD-1 also promotes metabolic rewiring in T cells^18,19^. However, how to modulate CD8^+^ T cell metabolism to sensitize them to anti-PD-1 treatment and enhance their effector function in solid tumours is still largely unknown.

Here, we performed an in vivo CD8^+^ T cell CRISPR screening in primary tumours, metastatic niches and secondary lymphoid organs complemented by single-cell RNA sequencing (scRNA-seq) to identify metabolic determinants governing CD8^+^ T cell functionality and enhancing responsiveness to anti-PD-1 treatment in solid refractory tumours. Our integrative approach unveiled the elongation of very long-chain fatty acid protein 1 (*Elovl1*), a gene encoding for a key enzyme for the synthesis of saturated very long-chain fatty acids (VLCFAs). Through mouse models of PDAC and melanoma, and ACT of antigen-specific CD8^+^ T cells, we demonstrate that *Elovl1* deficiency amplifies T cell antitumoural activity when combined with anti-PD-1 treatment. Mechanistically, in CD8^+^ T cells the reduction of saturated VLCFAs mediate INSIG1 destabilization, leading to increased cholesterol synthesis and uptake. The increased energy demand is fulfilled by augmented mitochondrial fitness and palmitate oxidation. Notably, in patients undergoing anti-PD-1 therapy, low *ELOVL1* expression in CD8^+^ tumour-infiltrating lymphocytes (TILs) correlated with a favourable treatment response. In conclusion, our targeted metabolic intervention not only enhances the memory pool of CD8^+^ T cells but also optimizes their effector polyfunctionality. This dual-action approach synergizes effectively with anti-PD-1 therapy, showcasing the potential to amplify its efficacy. In preclinical studies, our intervention demonstrates effectiveness against solid refractory tumours, making it a promising candidate for advancing therapeutic strategies in oncology.

## Results

### In vivo CRISPR screen identifies metabolic genes regulating CD8^+^ T cell fitness

**Fig. 1:**
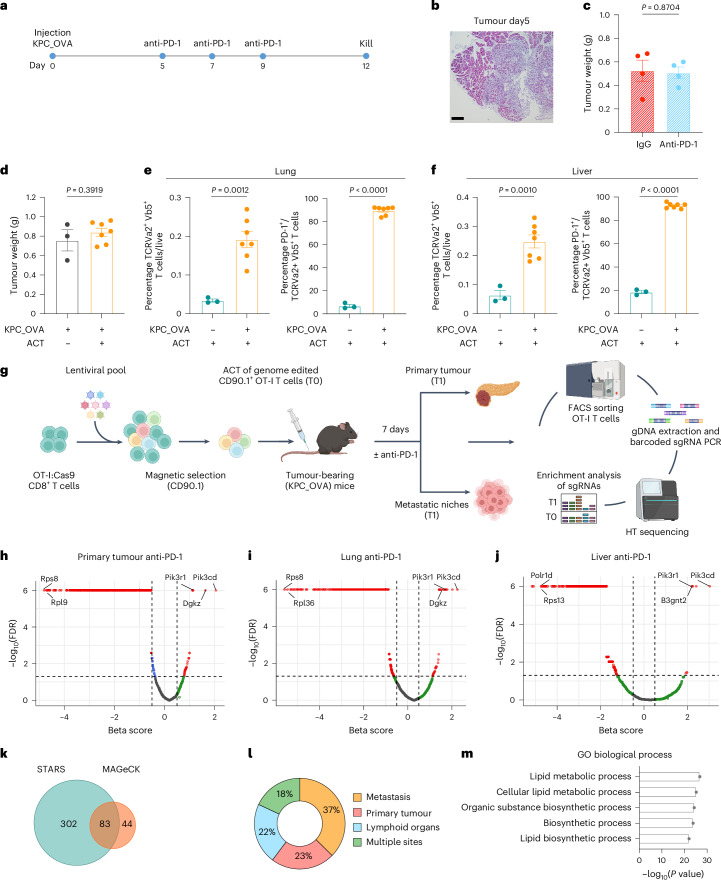
An in vivo CRISPR screen identifies metabolic genes regulating CD8^+^ T cell fitness in the tumour and metastatic organs. **a**–**c**, Experimental design of anti-PD-1 treatment resistance on KPC_OVA bearing mice (**a**); representative haematoxylin and eosin colouration of untreated KPC_OVA primary tumour, 5 days post-injection, scale bar 100 μM (**b**); KPC_OVA tumour weight at 12 days post-injection (**c**) (IgG-treated *n* = 4 versus anti-PD-1 *n* = 4). **d**, KPC_OVA tumour weights at 12 days with (*n* = 7) or without activated OT-I adoptive transfer (*n* = 3) at day 5 (ACT) (Extended Data Fig. 1a). **e**,**f**, Percentage of OVA-specific TCRvα2^+^ TCRvβ5^+^ T cells (left) and PD-1 expression (right) in lung (**e**) and liver (**f**) from naive or KPC_OVA-bearing mice receiving ACT (naive + ACT *n* = 3, KPC_OVA + ACT *n* = 7). **g**, Workflow of in vivo metabolic CD8^+^ T cells CRISPR/ Cas9 screening design from KPC_OVA bearing mice. Isolation and activation of OT-I T cells from OT-I:Rosa26-Cas9 mice; transduction of OT-I T cells with the lentiviral sgRNA metabolic library; Enrichment of transduced CD90.1^+^ OT-I; adoptive transfer of CD90.1^+^ OT-I T cells into recipient KPC_OVA-bearing mice and treatment with anti-PD-1 blocking antibody; 7 days post-ACT, sort of CD90.1^+^ OT-I cells from primary tumour, metastatic niches and lymphoid organs. NGS and bioinformatic identification of the candidate metabolic targets (IgG *n* = 41, anti-PD-1 *n* = 40, three independent sequencing experiments). Figure created in BioRender (Mazzone (2025) p04f031). **h**–**j**, Representative volcano plot generated with MAGeCK of enriched and depleted genes in CD90.1^+^ OT-I T cells sorted from primary tumour (**h**), lungs (**i**) and liver (**j**) of KPC_OVA tumour-bearing mice treated with anti-PD-1. **k**, Venn diagram representing the genes corresponding to the significantly enriched sgRNAs, at least in one organ and one treatment, using STAR (*P* ≤ 0.05) and MAGeCK (false discovery rate (FDR) ≤ 0.05) algorithms. **l**, Tissue distrubution of the 83 genes enriched in STAR and MAGeCK. **m**, Top five GO terms for the 83 genes enriched in STAR and MAGeCK. Data are presented as mean ± s.e.m. Statistical significance was assessed by two-tailed unpaired Student’s *t*-test. Source data

**Extended Data Fig. 1:**
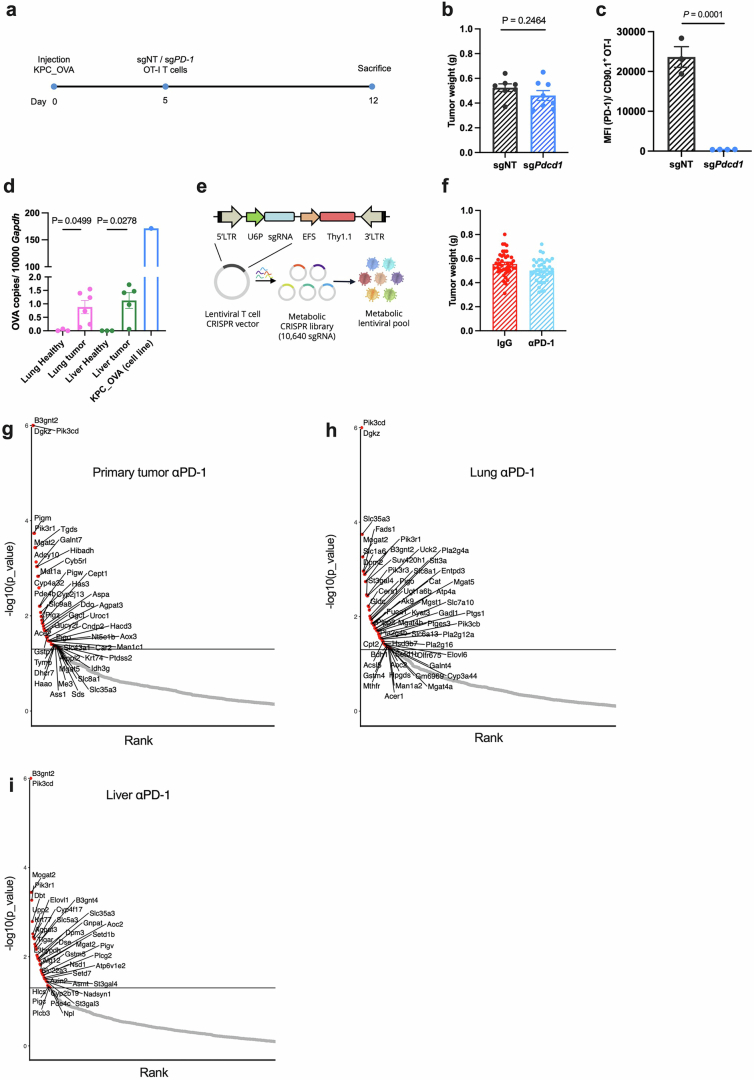
in vivo CRISPR screen identifies metabolic genes regulating CD8^+^ T cell fitness in PDAC. **a-c**, Schematics of PD-1 KO OT-I T cells treatment resistance validation (**a**); tumour weight at day 12 (**b**); PD-1 expression by flow cytometry in sg*Pdcd1* and sgNT OT-I T cells infiltrating the primary tumour (**c**) (**b**, sgNT n=7 sg*Pdcd1* =8; **c**, sgNT n=3 sg*Pdcd1* =4). **d**, KPC_OVA cancer cells quantification in metastatic organs (lung, liver) via RT-qPCR. KPC_OVA represents cultured KPC_OVA cells, as a positive control (Lung healthy n=3, tumour n=6; Liver healthy n=3, tumour n=5). **e**, Representation of metabolic library lentiviral vector. **f**, Tumour weight of mice injected with library transduced OT-I T cells and treated with control IgG (n=42) or anti-PD-1 (n=40). **g-i**, Representative volcano plot generated with STARS of targeted gene distribution in CD90.1^+^ OT-I T cells sorted from primary tumour (**g**), lungs (**h**) and liver (**i**) of KPC_OVA tumour-bearing mice treated with anti-PD-1. Data are presented as the mean ± s.e.m. Statistical significance was assessed by two-tailed unpaired Student’s t-test (**b-f**). Source data

To identify metabolic determinants of response to immunotherapy in immunologically cold tumours, we exploited a clinically relevant model of PDAC, which involves orthotopic injection of the KPC (*LSL-KrasG12D/+; LSL-Trp53R172H/+; Pdx-1-Cre*) cell line into the pancreas head of immunocompetent mice. This model recapitulates key features of human PDAC in terms of composition, metastases and poor response to both chemo- and immunotherapies^20^. To enable an in vivo CD8^+^ T cell screening, we engineered these KPC cells to constitutively express chicken ovalbumin (OVA), referred to as KPC_OVA. Upon orthotopic injection, we confirmed the resistance of this model to anti-PD-1 blocking antibody (Fig. 1a–c). T cell-based (Fig. 1d) and immune-checkpoint therapy resistance was further verified by ACT of activated OVA-specific CD8^+^ T cells (OT-I) wild type (WT) (sgNT) or PD-1 knockout (KO) (sg*Pdcd1*), 5 days after implanting KPC_OVA cells (Extended Data Fig. 1a). Treatment with either PD-1 KO or WT OT-I T cells failed to induce tumour reduction (Extended Data Fig. 1b,c). Together, these data indicate that additional immunosuppressive cues are engaged in the TME to limit CD8^+^ T cell activity even upon blockade of the PD-1–PD-L1 immunosuppressive axis, thus providing a representative model to perform an in vivo metabolic screening in CD8^+^ T cells.

As we wanted to explore T cell infiltration and fitness also in the metastatic niches, we validated the presence of KPC_OVA cancer cells in lungs and liver of tumour-bearing mice 12 days after tumour implantation (Extended Data Fig. 1d). This was also proved by the fact that upon OT-I T cell transfer, a higher percentage of OT-I T cells were found in the lung and liver from tumour-bearing, compared with healthy tumour-free mice that were also injected with activated OT-I T cells (Fig. 1d–f). Moreover, adoptively transferred OT-I and endogenous OVA-specific T cells infiltrating the lung and the liver of tumour-bearing mice expressed higher levels of PD-1, compared with OT-I T cells infiltrating the lung and liver of healthy mice, indicating that the presence of the ovalbumin antigen induced their activation (Fig. 1e,f). Altogether these data prove the presence of KPC_OVA cancer cells in the most common metastatic niches of PDAC, namely lung and liver, of tumour-bearing mice 12 days after orthotopic injection with KPC_OVA cells.

Next, to identify which metabolic pathways curb CD8^+^ T infiltration in PDAC under immunotherapy, we performed a CRISPR KO screen of 2,078 genes involved in cellular metabolism. These were selected by integrating the metabolic mouse genes from the Kyoto Encyclopaedia of Genes and Genomes with previously published high-quality reconstructions of mouse metabolism^21,22^. We next designed and generated a metabolic single guide RNA (sgRNA) library containing 10,390 gene-specific sgRNA and 250 nontargeting controls. This library was cloned into a lentiviral CRISPR vector that additionally contains a CD90.1 (Thy1.1) expression cassette to mark transduced T cells (Extended Data Fig. 1e). To perform the screen, we transduced OT-I Cas9 knock-in T cells with the lentiviral metabolic library and adoptively transferred them to KPC_OVA tumour-bearing mice. The same day we also started the treatment with anti-PD-1 or control antibody (Fig. 1g). Seven days after ACT, we killed the mice to sort sgRNA-transduced CD90.1^+^ OT-I T cells. We observed no difference in tumour size of mice that received anti-PD-1 or control treatment (Extended Data Fig. 1f).

To explore potential targets able to sustain T cell fitness in the primary tumour and in the metastatic niches, thus achieving a multi-organ effect, we sorted sgRNA-transduced CD90.1^+^ OT-I T cells from the primary tumour, the metastatic niches (lung, liver and peritoneal metastases), and the lymphoid organs (spleen, draining and nondraining lymph nodes) (Fig. 1g).

The sgRNA representation in the sorted T cells was determined by high-throughput sequencing and data were analysed with MAGeCK^23^. This enabled us to identify metabolic targets enriched in the different niches under a specific treatment condition (Fig. 1h–j and Supplementary Table 1). Among them, we retrieved sgRNAs targeting metabolic genes known to sustain T cells fitness and antitumoural activity such as *Dgkz*^24–26^, *Pi3k* family members^27–29^ and *B3gnt2* (ref. ^30^), whereas essential genes such as those encoding ribosomal protein L9 (*Rpl9*) or subunit D of RNA polymerase I and III (*Polr1d*), were significantly depleted (Fig.1h–j). The same analysis was performed with a second algorithm, STARS^31^(Extended Data Fig. 1g,h and Supplementary Table 1).

Of all genes targeted, 83 were found to be significantly enriched in at least one organ and treatment condition by both algorithms (Fig. 1k). When looking at their distribution among the different niches, most of them were particularly enriched in one niche, whereas 18% of these targets were retrieved from multiple sites (Fig. 1l). This suggests that different genes deletions might give a superior benefit in specific environments, but does not exclude relevance in other niches. With our approach, we aimed at identifying genes with enhanced activity in primary tumour and metastatic niches, thus achieving sustained CD8^+^ T cell persistence in vivo.

Last, to investigate the metabolic pathways specifically enriched among these 83 genes, we performed a Gene Ontology (GO) pathway analysis, which revealed lipid and small molecule metabolism as well as organic substance biosynthesis as the most represented biological processes (Fig. 1m). Altogether, these analyses validate our in vivo metabolic multi-organ CD8^+^ T cell screen and highlight 83 metabolic genes inhibiting T cell accumulation in the tumoural and lymphoid niches.

### In vivo CROP-seq screen positions *Elovl1* as a top metabolic target in CD8^+^ T cells

**Fig. 2:**
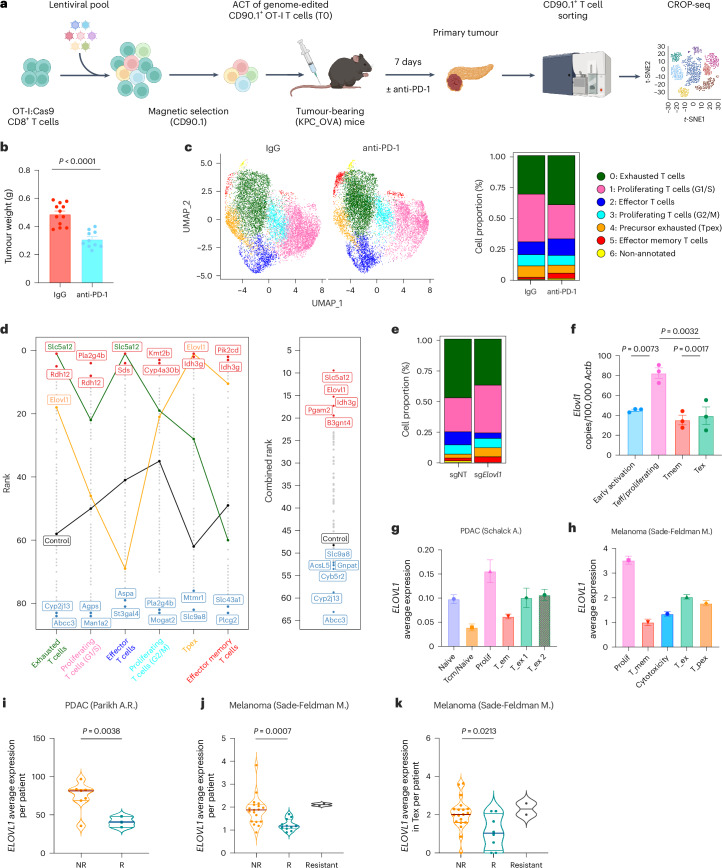
An in vivo single-cell CRISPR screen selects *Elovl1* as a promising metabolic target to sustain CD8^+^ T cell activity. **a**, Workflow of in vivo single-cell metabolic CD8^+^ T cells CRISPR/Cas9 screening design (CROP-seq) from KPC_OVA primary tumour (IgG *n* = 12, anti-PD-1 *n* = 10, two independent experiments). Figure created in BioRender.com/, Mazzone (2025) b69d891. **b**, Tumour weights at day 12 of KPC_OVA-bearing mice that received OT-I T cells transduced with CROP-seq library and were treated with IgG (*n* = 12) or anti-PD-1 (*n* = 10). Exact *P* value = 0.000008. **c**, Uniform Manifold Approximation and Projection (UMAP) plot (left) and corresponding bar plot (right) of the CD90.1^+^ OT-I cells from mice treated with IgG or anti-PD-1. **d**, Ranking of each gene per cluster upon anti-PD-1 (right). Combined ranking from CROP-seq analysis (left). Negative score was given for cluster 0 and a positive score was given for clusters 1–5. The top genes ranked higher for proliferation, effector and memory functions (cluster 1–5) and lower for exhaustion (cluster 0). **e**, Distribution of the clusters identified in **c** for *Elovl1*-deleted (sg*Elovl1*) or control OT-I (sgNT) under anti-PD-1 treatment. **f**, *Elovl1* expression quantified by RT–qPCR in in vitro differentiated CD8^+^ T cell states (*n* = 3, cells from three mice were used and kept separately as biological replicates). **g**,**h**, Average *ELOVL1* expression in different clusters of CD8^+^ TILs from single-cell datasets of patients with PDAC (**g**) and melanoma (**h**). **i**, *ELOVL1* expression per patient in CD8^+^ TILs of patients with PDAC showing primary resistance (nonresponders (NR) *n* = 7) or response (responders (R) *n* = 3) to anti-PD-1 treatment. **j**,**k**, *ELOVL1* expression per patient in total CD8^+^ TILs (**j**) and in effector/exhausted CD8^+^ TILs (**k**) of patients with melanoma showing primary resistance (NRs *n* = 19), response (R, *n* = 11) or acquired resistance to anti-PD-1 treatment (resistant *n* = 2). Data are presented as the mean ± s.e.m. Statistical significance was assessed by two-tailed unpaired Student’s *t*-test (**b**,**i**–**k**) or one-way analysis of variance (ANOVA) (**f**). Source data

**Extended Data Fig. 2:**
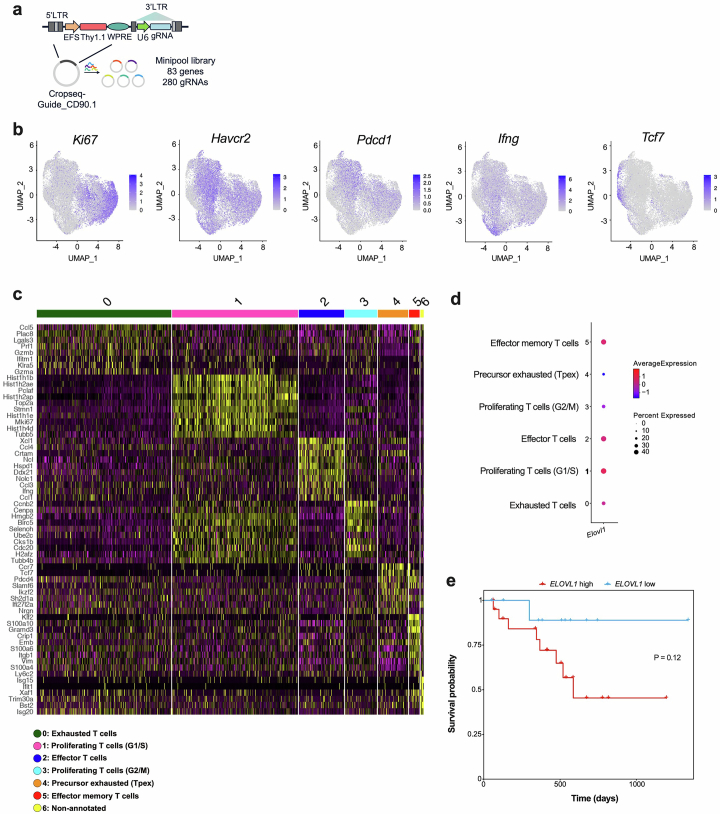
in vivo CROP-seq selects *Elovl1* as a promising target to sustain CD8^+^ T cell. **a**, Schematic representation of metabolic library CROP-seq lentiviral vector. **b**, UMAP showing the main gene characterizing the different clusters. **c**, Heatmap showing the most expressed genes in each cluster. **d**, Dot plot of *Elovl1* expression among the different clusters in the control OT-I T cells retrieved from the CROP-seq (control cells harbour the non-targeting guides of the CROP-seq library). **e**, Survival curve of melanoma patients treated with anti-PD-1 and having ELOVL1^low^ or ELOVL1^high^ CD8^+^ TILs (n=32). Statistical significance was assessed by unpaired two-tailed Student’s t-test (**e**). Source data

To prioritize and distil the most therapeutically relevant targets, we set out to characterize the phenotypic and functional heterogeneity associated with each metabolic gene, focusing on the primary tumour niche. To this aim, we combined our CRISPR screen with scRNA-seq (CROP-seq)^32^. A new set of sgRNAs targeting the distilled 83 metabolic genes was designed and cloned into a CROP-seq library (Extended Data Fig. 2a). The corresponding lentiviral library was transduced in OT-I T cells, which were used for adoptive cell transfer in mice bearing KPC_OVA pancreatic tumours and treated with anti-PD-1 or an IgG control antibody (Fig. 2a). Of note, we observed significantly smaller tumours in mice receiving OT-I T cells transduced with the CROP-seq metabolic library and treated with anti-PD-1 (Fig. 2b), a first indication that, among the distilled 83 genes, we successfully enriched candidate genes whose inhibition synergizes with anti-PD-1.

We then sorted sgRNA-transduced CD90.1^+^ OT-I T cells from the primary tumour and analysed the transcriptome and sgRNA expression of 22,371 T cells at single-cell resolution. Clustering analysis revealed seven different phenotypes characterizing OT-I T cells in PDAC primary tumour: cycling T cells (clusters 1 and 3), terminally exhausted T cells expressing inhibitory molecules (for example *Havcr2* and *Pdcd1*) (cluster 0), effector T cells expressing inflammatory cytokines (for example *Ifng* and *Tnfa*) (cluster 2), precursor exhausted T cells (Tpex) that express *Tcf7* and *Slamf6* (cluster 4)^33^ and effector memory T cells expressing *Klf2*, *Tcf7* and *Gzma* (cluster 5) (Extended Data Fig. 2b,c). T cells from anti-PD-1-treated animals differed in phenotype from control-treated animals, with more T cells having an effector phenotype (cluster 0, 2 and 5) and fewer showing a precursor exhausted phenotype (cluster 4), in line with previous studies^33^ (Fig. 2c). Next, we ranked the different gene knockouts by their CD8^+^ T cell phenotype, to pinpoint targets displaying sustained proliferation, more cytotoxicity, better memory and less exhaustion upon anti-PD-1 treatment and calculated a combined ranking for each target (Fig. 2d and Supplementary Table 1). *Slc5a12*, encoding the sodium-coupled monocarboxylate transported 2, ranked first in the combined rank, and was particularly enriched in effector T cells (cluster 2), where it ranked first as well. On the contrary, the second-ranking gene *Elovl1*, encoding the elongation of very long-chain fatty acid protein 1, ranked first in the cluster of Tpex T cells (cluster 4) (Fig. 2d). As the promotion of memory-like phenotypes is important for in vivo T cell persistence, we selected *Elovl1* for further validation. When looking at the phenotypic profile, *Elovl1*-depleted cells were also abundant in proliferation and showed a decrease in exhausted T cell populations (Fig. 2e). In the multi-organ screen, *Elovl1* resulted enriched particularly in the liver of mice treated with anti-PD-1 (Extended Data Fig. 1i) and ranked well in both the primary tumour and lungs of mice treated with anti-PD-1 (position 193 and 153, respectively; Supplementary Table 1), thus highlighting the efficiency of our approach in identifying putative targets having a systemic relevance and synergizing with anti-PD-1 treatment. As the role of *Elovl1* in CD8^+^ T cells is unknown, we first quantified its expression levels across different CD8^+^ T cell states. To do so, we collected T cells after 24 h of activation (early activation), after 72 h (effector/proliferative) and memory or exhausted differentiated CD8^+^ T cells. We found that *Elovl1* was mostly expressed by highly proliferating T cells (Fig. 2f), which require high amounts of lipids to sustain membrane generation and organelles biosynthesis and will, therefore, upregulate lipid synthesis pathways. *Elovl1* expression across different T cell clusters was also confirmed in vivo in the sgNT population from CROP-seq in our orthotopic KPC mouse model, (Extended Data Fig. 2d). To investigate the translatability of this target, we performed the same analysis on CD8^+^ TILs from human single-cell datasets of patients with PDAC^34^ or melanoma^35^. In line with our murine data, also in human patients, proliferating CD8^+^ TILs proved to be the cluster with the highest *ELOVL1* expression (Fig. 2g,h). Last, considering that in our screening, *Elovl1*-deficient CD8^+^ T cells were strongly enriched upon anti-PD-1 treatment, we wondered whether this was recapitulated in human patients. Therefore, we analysed *ELOVL1* expression in the same scRNA-seq dataset from patients with melanoma treated with anti-PD-1 (ref. ^35^) and in a bulk-RNA sequencing dataset of patients with PDAC treated with anti-PD-1 and anti-CTLA-4 in combination with radiation^36^ (given the absence of scRNA sequencing PDAC datasets similar to those found in patients with melanoma where ICB-based therapy is the first-line treatment option).

CD8^+^ TILs showed significantly lower *ELOVL1* expression in both patients with PDAC and melanoma responding to anti-PD-1 therapy compared with nonresponding or resistant patients (Fig. 2i,j). Furthermore, in scRNA sequencing of melanoma, exhausted/effector CD8^+^ TILs showed significantly lower *ELOVL1* expression in responders than in nonresponders and resistant patients (Fig. 2k), suggesting that *ELOVL1*^low^ TILs are more effective in synergizing with anti-PD-1 treatment than *ELOVL1*^high^ TILs. This was also reflected in patient survival, as anti-PD-1-treated patients with melanoma and *ELOVL1*^low^ TILs showed better overall survival than anti-PD-1-treated patients with melanoma and *ELOVL1*^high^ TILs, albeit borderline significantly (Extended Data Fig. 2e).

Together these data highlight *Elovl1* as a relevant metabolic target with a strong translational potential.

### *Elovl1-*deficient CD8^+^ T cells increase antitumoural activity upon anti-PD-1

**Fig. 3:**
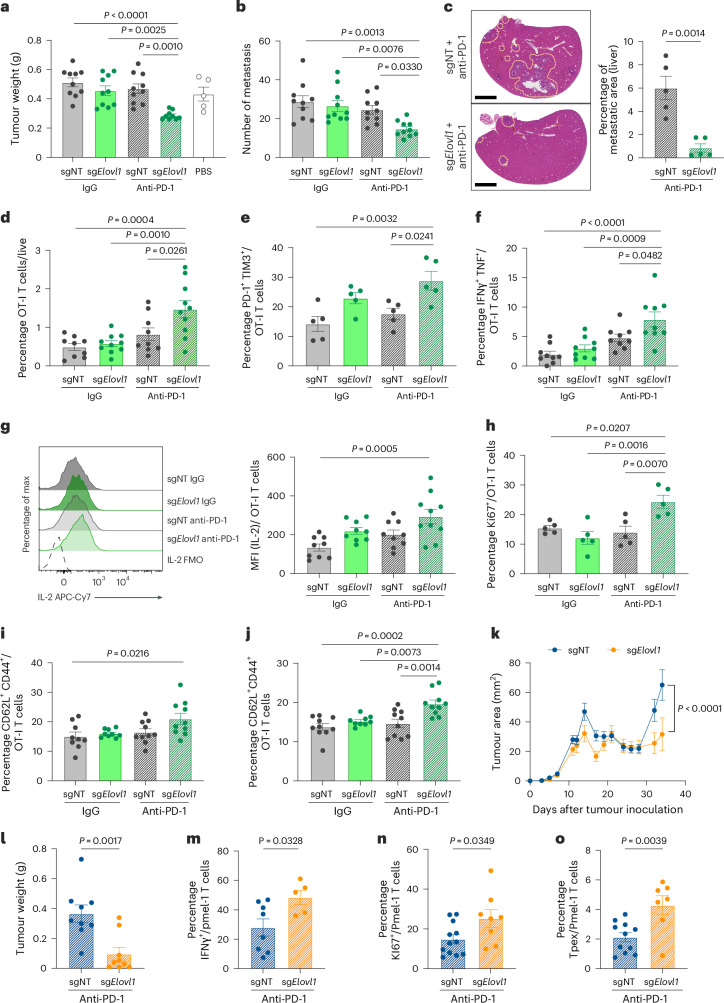
*Elovl1*-deficient CD8^+^ T cells have increased antitumoural activity upon anti-PD-1 treatment. **a**,**b**, Tumour weight (**a**) and peritoneal metastasis count (**b**), on day14 post-KPC_OVA injection, of mice not treated (PBS), receiving sgNT or sg*Elovl1* OT-I T cells and treated with IgG or anti-PD-1. **c**, Representative haematoxylin and eosin picture of one lobe of the liver (scale bar, 2.5 mm, left) and percentage of tumour area (right) in an experimental model of PDAC liver metastasis. The mice were injected intrasplenic with KPC_OVA. Eight days later, they received sgNT or sg*Elovl1* OT-I T cells and started anti-PD-1 therapy the following day (*n* = 5). Tumour burden was quantified on every lobe and calculated as percentage of total area. **d**, Flow cytometry quantification of sgNT and sg*Elovl1* OT-I cells in the primary tumour of mice treated with IgG or anti-PD-1. **e**,**f**, Flow cytometry quantification of PD-1^+^ Tim3^+^ (**e**) and TNF^+^ IFNγ^+^ (**f**) percentage of sgNT and sg*Elovl1* OT-I cells infiltrating KPC_OVA primary tumour of mice treated with IgG or anti-PD-1. **g**, Representative flow cytometry histogram and quantification of IL-2 production (median of fluorescence intensity; MFI) of sgNT or sg*Elovl1* OT-I T cells infiltrating KPC_OVA primary tumour of mice treated with IgG or anti-PD-1. **h**–**j**, Flow cytometry quantification of Ki67 (**h**) and CD44^+^CD62L^+^ (Tcm) (**i**) in sgNT or sg*Elovl1* OT-I T cells infiltrating the spleen and CD44^+^ CD62L^+^ (Tcm) in sgNT or sg*Elovl1* OT-I cells infiltrating the draining lymph nodes (**j**) of KPC_OVA-bearing mice treated with IgG or anti-PD-1. **k**, Representative tumour growth curve of mice injected subcutaneously with B16F1 cells and treated with anti-PD-1 that received sgNT or sg*Elovl1* pmel-1 T cells (sgNT *n* = 8; sg*Elovl1* *n* = 5). **l**, Tumour weight of B16F1 tumour-bearing mice injected with sgNT or sg*Elovl1* pmel-1 cells (sgNT *n* = 12, sg*Elovl1* *n* = 11). **m**,**n**, Flow cytometry quantification of IFNγ^+^ (**m**) and Ki67^+^ (**n**) pmel-1 T cells infiltrating B16F1 primary tumour (sgNT *n* = 8, sg*Elovl1* *n* = 5). **o**, Flow cytometry quantification of PD-1^+^ Slamf6^+^ (Tpex) pmel-1 T cells infiltrating B16F1 primary tumour (sgNT *n* = 11, sg*Elovl1* *n* = 7). (**a**,**b**,**f**,**g**,**I**,**j**, sgNT + IgG, *n* = 10; sg*Elovl1* + IgG, *n* = 10, sgNT + anti-PD-1, *n* = 10; sg*Elovl1* + anti-PD-1, *n* = 10, two independent experiments). (**e**,**h**, sgNT + IgG, n = 5; sg*Elovl1* + IgG, *n* = 5, sgNT+ anti-PD-1, *n* = 5; sg*Elovl1* + anti-PD-1, *n* = 5). Data are presented as mean ± s.e.m. Statistical significance was assessed by one-way (**a**,**b**,**d**–**j**) or two-way (**k**) ANOVA and by two-tailed unpaired Student’s *t*-test (**c**,**l**–**o**). Source data

**Extended Data Fig. 3:**
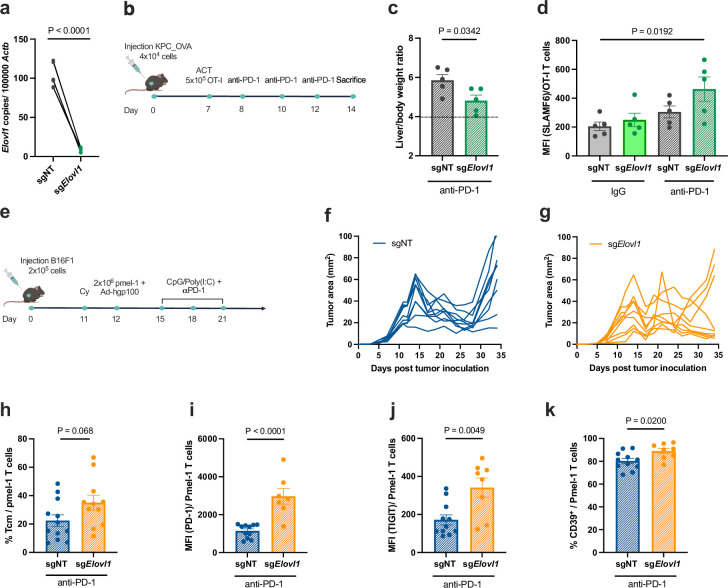
*Elovl1*-deficient CD8^+^ T cells increased activity upon anti-PD-1 treatment. **a**, *Elovl1* expression assessed by qRT-PCR (n=7) in vitro in sgNT or sg*Elovl1* OT-I T cells at day 7 from activation. Exact p value = 0.000001. **b**, Experimental design of *Elovl1* in vivo validation in PDAC model. **c**, Liver to body weight ratio in an experimental model of PDAC liver metastasis. The mice were injected intrasplenic with KPC_OVA. Eight days later, they received sgNT or sg*Elovl1* OT-I T cells and started anti-PD-1 therapy the following day (n=5). **d**, SLAMF6 expression on OT-I T cells infiltrating PDAC primary tumour (n=5). **e**, Schematics of pmel-1 T cells ACT experiment. **f, g**, Tumour growth spider plots in B16F1 tumour-bearing mice injected with sgNT (**f**) or sg*Elovl1* (**g**) pmel-1 T cells (sgNT n=12, sg*Elovl1* n=11). **h**, Flow cytometry quantification of CD44^+^ CD62L^+^ (Tcm) in pmel-1 T cells infiltrating the spleen of B16F1-bearing mice (sgNT n=12, sg*Elovl1* n=11, two independent experiments). **i**–**k**, Flow cytometry quantification of PD-1 (**i**), TIGIT (**j**) and CD39 (**k**) in pmel-1 T cells infiltrating the primary tumour of B16F1-bearing mice (sgNT n=11, sg*Elovl1* n=8). (**i**) Exact p value = 0.000095. Data are presented as the mean ± s.e.m. Statistical significance was assessed by paired (**a**) or unpaired (**c, h–k**) two-tailed Student’s t-test and one-way ANOVA (**d**). Parts of this figure were created in BioRender. Mazzone, M. (2025) https://BioRender.com/ s90t255. Source data

To validate the impact of *Elovl1* inactivation on the antitumoural activity of CD8^+^ T cells in PDAC, we deleted *Elovl1* in in vitro-activated OT-I T cells by nucleofection of a gRNA targeting *Elov1* (sg*Elovl1*) or a nontargeting gRNA (sgNT) in complex with Cas9 (Extended Data Fig. 3a). Seven days after the orthotopic injection of KPC_OVA cells, we performed adoptive transfer of sg*Elovl1* or sgNT OT-I T cells and initiated anti-PD-1 or control treatment (Extended Data Fig. 3b). This significantly reduced the tumour weight as well as the number of peritoneal metastases in mice treated with sg*Elovl1* OT-I T cells and anti-PD-1, but not in mice receiving anti-PD-1 or sg*Elovl1* OT-I T cells alone (Fig. 3a,b).

In our multi-organ screening, *Elovl1* was significantly enriched in the liver of mice treated with anti-PD-1 (Extended Data Fig. 1i). Therefore, we performed ACT of sgNT or sg*Elovl1* OT-I T cells in an experimental model of PDAC liver metastasis. The mice receiving sg*Elovl1* OT-I T cells in combination with anti-PD-1 showed a reduced tumour burden (Fig. 3c and Extended Data Fig. 3c).

Together, these data indicate that *Elovl1* deletion in CD8^+^ T cells synergizes with anti-PD-1 treatment to overcome immunotherapy resistance in our pancreatic cancer model.

Considering that we performed CROP-seq in the primary tumour, we further characterized the impact of *Elovl1*-deficient OT-I T cells in the same tissue. In line with our screening, we observed more sg*Elovl1* OT-I T cells infiltrating anti-PD-1-treated tumours compared with control conditions (Fig. 3d).

When analysing the in vivo phenotype, *Elovl1*-deficient OT-I T cells expressed more co-inhibitory molecules such as PD-1 and TIM-3 in the presence of anti-PD-1 treatment, compared with the control OT-I T cells (Fig. 3e). Despite the exhausted phenotype, they retained higher, production of IFNγ, TNF and IL-2 (Fig. 3f,g). Additionally, sg*Elovl1* OT-I T cells expressed higher Ki67, validating the increase in proliferative capacity hinted by CROP-seq (Fig. 3h). In conclusion, genetic ablation of *Elovl1* combined with anti-PD-1 treatment improves CD8^+^ T cell activation and functionality in the immune-suppressive microenvironment of pancreatic cancer.

Moreover, the cluster of Tpex was expanded in sg*Elovl1* compared with sgNT OT-I T cells (Fig. 2e). By flow cytometry we validated the higher expression of Slamf6, a key Tpex marker, in sg*Elovl1* OT-I T cells infiltrating anti-PD-1-treated tumours compared with control groups (Extended Data Fig. 3d).

As Tpex are known to endow memory T cell features, we also investigated the role of *Elovl1* in the generation of other memory populations. In both draining lymph nodes and spleen, anti-PD-1 treatment and sg*Elovl1* inactivation synergized to produce more CD62L^+^ CD44^+^ central memory T cells (Tcm) (Fig. 3i,j). Considering that anti-PD-1 is widely used for the treatment of melanoma patients, we confirmed these observations also in B16F1 tumours, by transferring CD8^+^ T cells specific for gp100 (pmel-1), an endogenously expressed melanocytic lineage antigen (Extended Data Fig. 3e). When performing anti-PD-1 treatment alongside ACT of sg*Elovl1* pmel-1 T cells, we observed a delay in tumour growth (Fig. 3k and Extended Data Fig. 3f,g), reflected by a lower tumour weight (Fig. 3l), compared with sgNT pmel-1 T cell administration. Like in the KPC model, sg*Elovl1* pmel-1 T cells infiltrating a melanoma primary tumour were more functional with higher IFNγ production (Fig. 3m) and proliferative potential (Fig. 3n) compared with control T cells. Moreover, *Elovl1*-deficient pmel-1 T cells showed higher Tpex and Tcm differentiation in the tumour and spleen, respectively (Fig. 3o and Extended Data Fig. 3h), as well as increased expression of co-inhibitory modules (Extended Data Fig. 3i–k). Together, these data show that *Elovl1* deletion rewires CD8^+^ T cells phenotype allowing their reinvigoration by anti-PD-1, thus leading to improved functions. In conclusion, this therapeutic combination can mediate reduced tumour growth in different cancer types.

### *Elovl1*-deficient CD8^+^ T cells show a rewired lipid profile

**Fig. 4:**
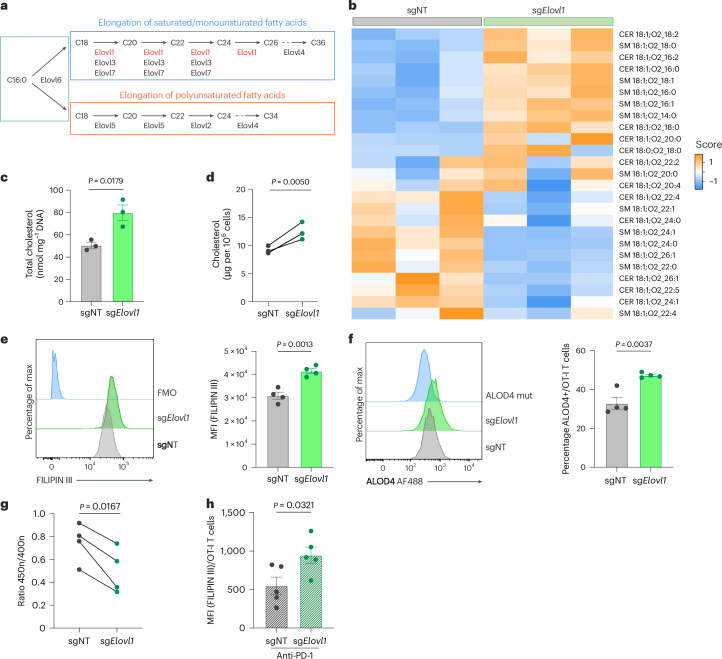
*Elovl1*-deficient CD8^+^ T cells show a rewired lipid profile and an increase in cholesterol levels. **a**, Schematics of ELOVL family activity. **b**,**c**, LS-MS lipidomics on in vitro sgNT and sg*Elovl1* OT-I T cells (*n* = 3). Heatmap representing the relative abundance of each specific lipids (**b**) and quantification of total cholesterol (**c**). **d**, Total cellular cholesterol quantification via Amplex Red cholesterol kit in in vitro sgNT or sg*Elovl1* OT-I T cells (*n* = 3). **e**, Membrane cholesterol quantification with FILIPIN III (*n* = 4). Representative histogram (left) and MFI quantification (right) in in vitro sgNT or sg*Elovl1* OT-I T cells. **f**, Flow cytometry quantification of plasma membrane cholesterol with AlexaFluor 647 conjugated ALOD4 in cultured sgNT and sg*Elovl1* OT-I T cells (*n* = 3). Representative histogram (left) and quantification (right). **g**, Flow cytometry quantification of the membrane fluidity using the fluorescent probe PDA in vitro in sgNT or sg*Elovl1* OT-I T cells. Data are shown as excimer/monomer ratio (450 nm/400 nm) (*n* = 4). **h**, Membrane cholesterol quantification with FILIPIN III (*n* = 5) in sgNT or sg*Elovl1* OT-I T cells infiltrating KPC_OVA tumours of mice treated with anti-PD-1. Data are presented as the mean ± s.e.m. Statistical significance was assessed by two-tailed unpaired (**c**,**e**,**f**,**h**) and paired (**g**,**d**) Student’s *t*-test. Source data

**Extended Data Fig. 4:**
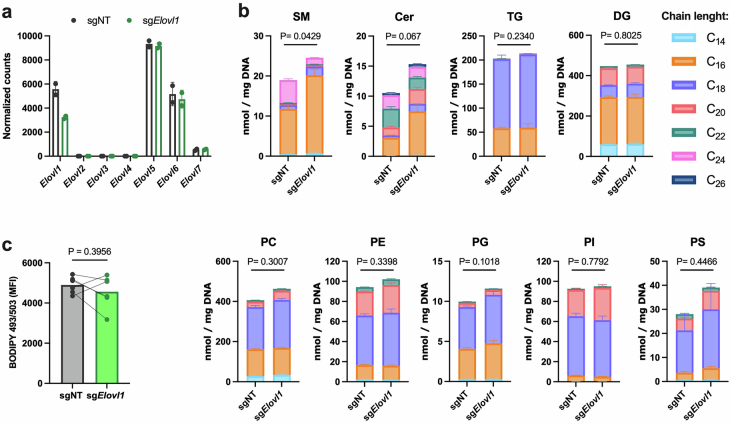
*Elovl1*-deficient CD8^+^ T cells show a rewired lipid profile. **a**, *Elovl1-7* expression quantified by in vitro bulk-RNA sequencing of sgNT and sg*Elovl1* OT-I T cells at day 7 from activation. The histogram shows the normalized read counts of *Elovl* family members. **b**, LS-MS lipidomics (n=5). Quantification of total lipid species. For each lipid species, the contribution of fatty acids with different chain lengths is shown. The statistic is applied to the total quantity of lipids in each species. SM= sphingomyelins; Cer= Ceramides; TG= triacylglycerides; DG= diacylglycerides; PC= phosphatidylcholine; PE= phosphatidylethanolamine; PG= phosphatidylglycerol; PI= phosphatidylinositol; PS= phosphatidylserine. **c**, Quantification of lipid droplets by the fluorescent probe BODIPY 493/503. Cells were incubated with the probe and MFI was quantified by flow cytometry (n=6). Data are presented as the mean ± s.e.m. Statistical significance was assessed by unpaired (**b**) or paired (**c**) two-tailed Student’s t-test. Source data

ELOVL1 is one of seven enzymes that elongate VLCFAs. Together with ELOVL3 and ELOVL7, ELOVL1 generates saturated and monounsaturated VLCFAs, whereas ELOVL2 and ELOVL5 generate polyunsaturated VLCFAs^37^ (Fig. 4a). As shown by the in vitro RNA sequencing of sgNT and sg*Elovl1* OT-I T cells (performed 7 days after activation), *Elovl1* deletion did not cause any compensatory upregulation of the other *Elovl* family members (Extended Data Fig. 4a). Considering its role in fatty acid elongation, we next evaluated the lipid composition of in vitro-activated CD8^+^ T cells nucleofected with sgNT or sg*Elovl1* using unbiased lipidomics. This revealed a generalized change across various lipid species (Extended Data Fig. 4b). In particular, we observed fewer lipids containing VLCFAs with chain lengths between 22 and 26 carbons (C_22_–C_26_) and more lipids with shorter fatty acid chains (C_16_–C_18_) (Fig. 4b and Extended Data Fig. 4b), thus functionally validating *Elovl1* deficiency. Our lipidomic analysis also revealed an increase in important constituents of plasma membrane microdomains, including sphingomyelins, ceramides (Extended Data Fig. 4b) and total cholesterol (Fig. 4c) in sg*Elovl1* compared with control CD8^+^ T cells. An orthogonal method also confirmed the increase in total cholesterol (Fig. 4d). Considering the profound lipid rewiring occurring in *Elovl1*-deficient CD8^+^ T cells, we wondered whether lipid storage would be also altered. Flow cytometry analysis of the neutral BODIPY 493/503, a fluorescent lipid binding to intracellular lipid droplets (LDs), showed a similar quantity in both control and *Elovl1*-deficient CD8^+^ T cells (Extended Data Fig. 4c).

Elevated free cholesterol at the plasma membrane is of particular interest, as it was previously associated with stronger T cell receptor (TCR) signalling^38^ and with a more potent effector phenotype of intratumoural CD8^+^ T cells^39^. To assess whether sg*Elovl1* CD8^+^ T cells also increased cholesterol in the cell membranes, we used FILIPIN III, a probe that binds only to free cholesterol^40^, and an AF488-conjugated Anthrolysin O domain 4 (ALOD4) recombinant protein, staining selectively the cholesterol at the plasma membrane^41^. This showed higher free cholesterol levels in cultured *Elovl1*-deficient CD8^+^ T cells (Fig. 4e,f), resulting in reduced plasma membrane fluidity (Fig. 4g). Elevated free cholesterol was also maintained in sg*Elovl1* CD8^+^ T cells infiltrating KPC_OVA tumours of mice treated with anti-PD-1 (Fig. 4h). In conclusion, deletion of *Elovl1* results in reduced incorporation of VLCFAs in most lipid species and in an accumulation of free cholesterol.

### INSIG1 degradation and SREBP2 activation mediate cholesterol increase in *Elovl1*-deficient CD8^+^ T cells

**Fig. 5:**
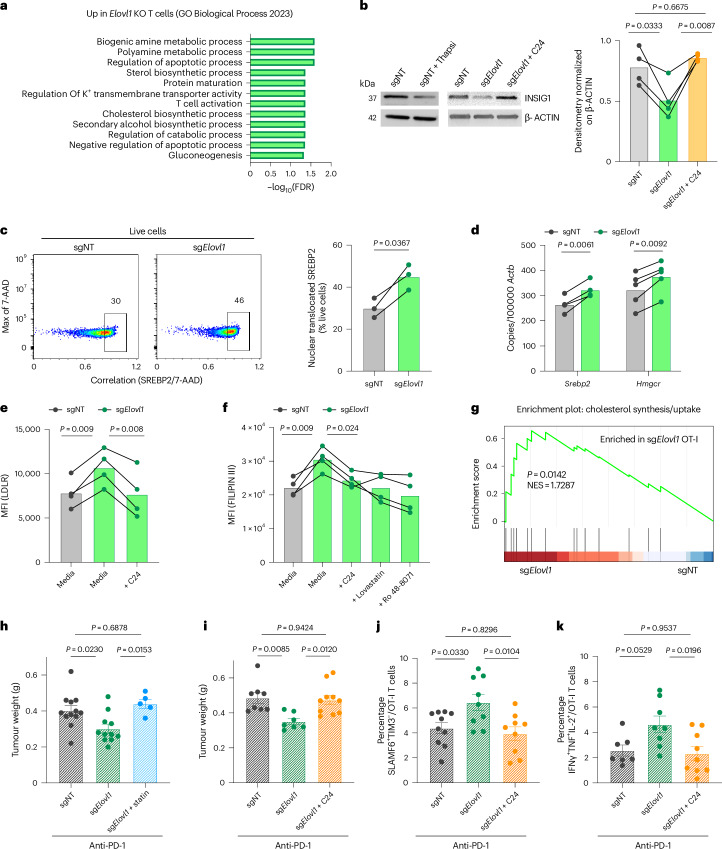
VLCFA reduction mediates INSIG1 instability and SREBP2 activation. **a**, Bulk-RNA sequencing analysis of biological process significantly upregulated in vitro in sg*Elovl1* (*n* = 2) compared with sgNT (*n* = 2) OT-I T cells on day 7 from activation (day of ACT). **b**, Representative western blot (left) and quantification (right) of INSIG1 in sgNT, sg*Elovl1* and sg*Elovl1* supplemented with 20 µM C24 OT-I T cells in vitro (*n* = 4) on day 5 from activation. **c**, Representative flow cytometry plots and quantification (correlation SREBP2/ 7-AAD) of SREBP2 nuclear translocation in sgNT or sg*Elovl1* OT-I T cells in vitro (*n* = 3) on day 5 from activation performed by BD FACSDiscover S8 Cell Sorter. **d**, *Srebp2* and *Hmgcr* expression assessed by RT–qPCR in vitro in sgNT or sg*Elovl1* OT-I T cells (*n* = 5) on day 5 from activation. **e**,**f**, in vitro flow cytometry quantification of LDLR (**e**) and FILIPIN III (**f**) in sgNT and sg*Elovl1* OT-I T cells ± lignoceric acid 20 µM (C24), 2 µM Lovastatin or 2 µM R0 48-8071 (*n* = 4) on day 5 from activation for 24 h. **g**, GSEA of cholesterol biosynthesis and uptake gene expression evaluated via bulk-RNA sequencing of sgNT (*n* = 3) or sg*Elovl1* (*n* = 3) OT-I T cells sorted from PDAC primary tumour of mice treated with anti-PD-1. **h**, Tumour weight on day14 post-KPC_OVA injection, of mice that received sgNT (*n* = 12), sg*Elovl1* (*n* = 11) or sg*Elovl1* pretreated with 2 µM Lovastatin (*n* = 5) OT-I T cells and treated with anti-PD-1. **i**, Tumour weight on day14 post-KPC_OVA injection, of mice that received sgNT (*n* = 8), sg*Elovl1* (*n* = 7) or sg*Elovl1* pretreated with 20 µM C24 (*n* = 10) OT-I T cells and treated with anti-PD-1 (two independent experiments). **j**,**k**, Flow cytometry quantification of SLAMF66^+^TIM3^−^ (**j**, Tpex) and IFNγ^+^TNF^+^IL-2^+^ (**k**, T polyfunctional) in sgNT, sg*Elovl1* or sg*Elovl1* pretreated with 20 µM C24 OT-I T cells infiltrating the primary tumour of KPC_OVA-bearing mice treated with anti-PD-1 (sgNT *n* = 10, sg*Elovl1* *n* = 9, sg*Elovl1* + C24 *n* = 9, two independent experiments). Data are presented as mean ± s.e.m. Statistical significance was assessed by paired (**c**,**d**) two-tailed Student’s *t*-test or one-way ANOVA (**b**,**f**,**h**–**k**). NES, normalized enrichment score. Source data

**Extended Data Fig. 5:**
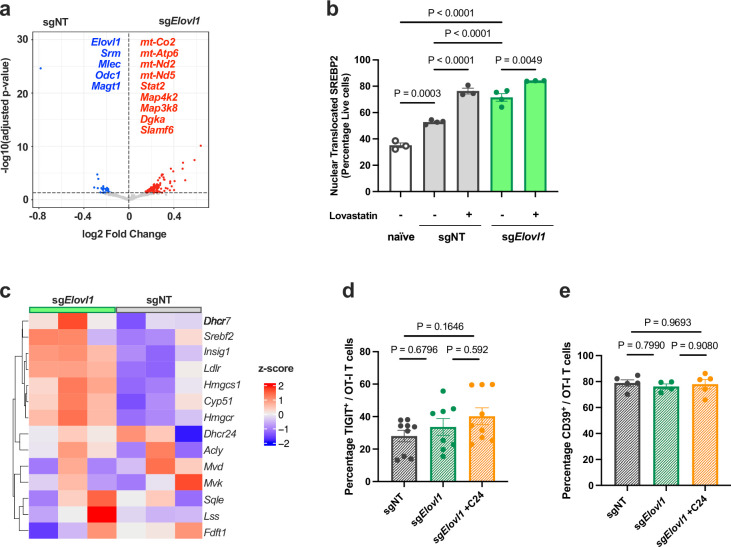
VLCFA reduction mediates INSIG1 instability and SREBP2 activation. **a**, Volcano plot of differentially expressed genes obtained by bulk-RNA sequencing of in vitro sgNT and sg*Elovl1* OT-I T cells at day 7 from activation (n=2). **b**, Flow cytometry quantification (correlation SREBP2/ 7-AAD) performed by BD FACSDiscover S8 Cell Sorter of SREBP2 nuclear translocation in vitro in sgNT and sg*Elovl1* OT-I T cells (n=4) at day 7 from activation. To validate the technique, we included naive CD8^+^ T cells as a negative control (n=3) and activated OT-I T cells treated with 2 µM Lovastatin (HMGCR inhibitor) as a positive control for SREBP2 translocation. Column B vs C p value = 0.000016; column B vs D p value = 0.000073. **c**, Heatmap showing the expression of cholesterol biosynthesis and uptake genes in sgNT (n=3) and sg*Elovl1* (n=3) OT-I T cells sorted, seven days after ACT, from PDAC primary tumour of mice treated with anti-PD-1. **d, e**, Flow cytometry quantification of TIGIT (**d**) and CD39 (**e**) in sgNT, sg*Elovl1* or sg*Elovl1* treated with 20 µM C24 infiltrating the primary tumour of KPC_OVA bearing mice treated with anti-PD-1 (**d**, sgNT n=9, sg*Elovl1* n=8, sg*Elovl1*+C24 n=9; **e**, sgNT n=5, sg*Elovl1* n=4, sg*Elovl1*+C24 n=5). Data are presented as the mean ± s.e.m. Statistical significance was assessed by one-way ANOVA (**b, d, e**). Source data

To better understand which pathways were affected by the deletion of *Elovl1* in CD8^+^ T cells we interrogated our in vitro RNA sequencing dataset, which showed cholesterol biosynthesis as one of the top upregulated biological processes in sg*Elovl1* compared with sgNT CD8^+^ T cells (Fig. 5a and Extended Data Fig. 5a).

Diverse sources of free cholesterol could explain its increase, including de novo biosynthesis and augmented uptake from the extracellular milieu. In CD8^+^ T cells as well as other cell types, sterol regulatory element-binding protein 2 (SREBP2) is the main transcription factor regulating these pathways. The activation of SREBP2 begins with the degradation of insulin-induced gene 1 (INSIG1), which enables the translocation of the SREBP2 and cleavage activating protein (SCAP) complex from the endoplasmic reticulum (ER) to the Golgi apparatus. There, a protease cleaves SREBP2, leading to its nuclear translocation. In yeast it has been demonstrated that an accumulation of saturated VLCFAs in the ceramides of the ER membrane prevents the ER-associated protein degradation (ERAD) complex from degrading INSIG1, thus leading to cholesterol shortage^42^. We measured INSIG1 protein levels through western blot analysis. Twenty-four hours following the genetic deletion of *Elovl1*, CD8^+^ T cells displayed markedly reduced INSIG1 levels compared with control cells. This reduction was similar to the effect observed in sgNT T cells treated with thapsigargin, a known inducer of ER-associated degradation (Fig. 5b). However, the supplementation of lignoceric acid (C24:0) to *Elovl1*-deficient T cells prevented INSIG1 degradation, suggesting that saturated VLCFAs play a role in regulating INSIG1 also in mammals (Fig. 5b).

In line with degraded INSIG1, *Elovl1*-deficient CD8^+^ T cells showed an increase in SREBP2 nuclear translocation (Fig. 5c and Extended Data Fig. 5b) and messenger RNA expression (Fig. 5d). We also confirmed higher expression levels in sg*Elovl1* CD8^+^ T cells of SREBP2 target genes HMG-CoA Reductase (*Hmgcr*), a rate-limiting cholesterol biosynthesis enzyme (Fig. 5d), and of the low-density lipoprotein receptor (LDLR) on the plasma membrane, important for cholesterol uptake (Fig. 5e). As for INSIG1, supplementation in the culture medium of C24:0, rescued the loss of *Elovl1*, with LDLR and free cholesterol levels normalizing to those found in control CD8^+^ T cells (Fig. 5e,f). Similar results were obtained when blocking cholesterol synthesis by using the HMGCR inhibitor lovastatin, or using Ro 48-8071, targeting 2,3-oxidosqualene cyclase (OSC), a distal enzyme in cholesterol biosynthesis (Fig. 5f). The cholesterol synthesis/uptake pathway was also enriched in the in vivo bulk-RNA sequencing analysis of sg*Elovl1* versus sgNT OT-I T cells isolated 7 days post-ACT, from PDAC primary tumours of mice treated with anti-PD-1 (Fig. 5g and Extended Data Fig. 5c).

Last, to confirm a direct role of VLCFAs and cholesterol in the phenotype modulations occurring in *Elovl1*-deficient CD8^+^ T cells, we performed ACT of sg*Elovl1* OT-I T cells pretreated with lignoceric acid or lovastatin in KPC_OVA tumour-bearing mice and treated them with anti-PD-1. Pretreatment with C24:0 or lovastatin prevented the beneficial effect mediated by *Elovl1* deletion on tumour growth control (Fig. 5h,i). Accordingly, upon lignoceric acid pretreatment, Tpex differentiation and polyfunctionality were also normalized to the level of sgNT CD8^+^ T cells (Fig. 5j,k), whereas the expression levels of TIGIT and CD39, both exhaustion markers, remained unchanged (Extended Data Fig. 5d,e).

In conclusion, saturated VLCFAs contribute to the stabilization of INSIG1, preventing cholesterol accumulation and hampering CD8^+^ T cell functions.

### *Elovl1* inhibition enhances TCR signalling and T cell activation

**Fig. 6:**
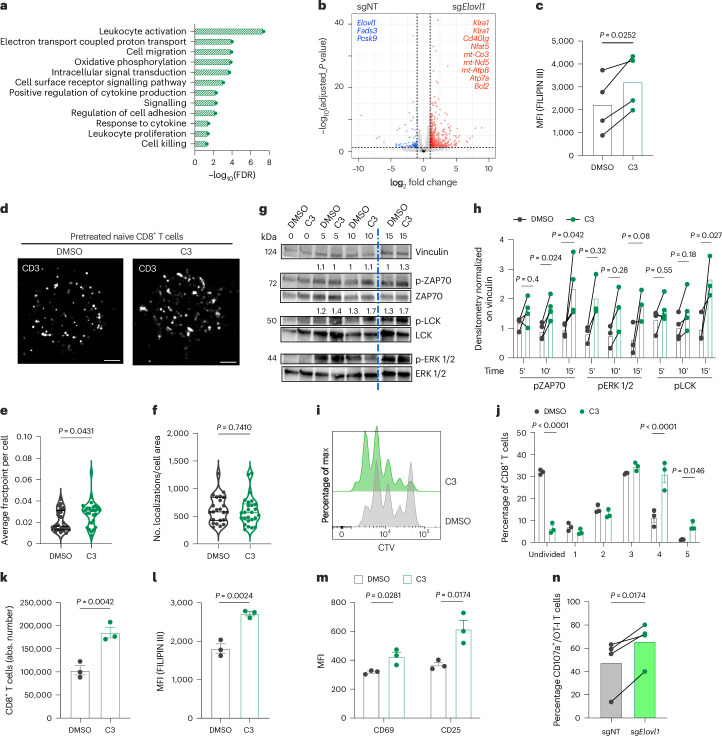
*Elovl1*-deficient CD8^+^ T cells present stronger TCR signalling and activation. **a**, Bulk-RNA sequencing analysis of biological process significantly upregulated in vivo in sg*Elovl1* compared with sgNT OT-I sorted, 7 days after ACT, from PDAC primary tumour of mice treated with anti-PD-1 (sgNT *n* = 3; sg*Elovl1* *n* = 3). **b**, Volcano plot of differentially expressed genes obtained by bulk-RNA sequencing of sgNT or sg*Elovl1* OT-I T cells sorted, 7 days after ACT, from PDAC primary tumour of mice treated with anti-PD-1 (sgNT *n* = 3; sg*Elovl1* *n* = 3). **c**, In vitro flow cytometry quantification of FILIPIN III in naive CD8^+^ T cells 6 h after treatment with DMSO or C3 (ELOVL1 inhibitor) (*n* = 4). **d**,**e**, Representative reconstructed dSTORM images (scale bar, 1 µm) (**d**) and mean fraction of clustered localizations (fractpoint) (**e**) in DMSO- and C3-treated naive murine CD8^+^ T cells (DMSO *n* = 22; C3 *n* = 26; four independent experiments). **f**, Quantification of total number of single localizations acquired per cell via dSTORM and normalized on cell area in DMSO- and C3-treated naive murine CD8^+^ T cells (DMSO *n* = 22; C3 *n* = 26; four independent experiments). **g**,**h**, Representative western blot (**g**) and quantification (**h**) of the main mediator of TCR signalling in naive CD8^+^ T cells pretreated for 6 h with DMSO or C3 and then activated with anti-CD3/anti-CD28 antibodies (*n* = 4, four independent experiments). The blue line indicates samples of the same experiment loaded on a separate gel (**g**). **i**–**k**, Representative flow cytometry graph of cell trace violet (CTV) dilution (**i**), quantification of cell in each division (**j**) and absolute count (**k**) of CD8^+^ T cells treated with DMSO or C3 and stimulated with anti-CD3/anti-CD28 activation beads (*n* = 3). **l**,**m**, Flow cytometry quantification of FILIPIN III (**l**), CD69 and CD25 (MFI) (**m**) in wild-type (WT) CD8^+^ T cells treated or not with C3, at 48 h post-activation with anti-CD3/anti-CD28 beads (*n* = 3). **n**, Flow cytometry quantification of CD107a^+^ sgNT or sg*Elovl1* OT-I T cells upon 5 h in vitro re-stimulation via KPC_OVA co-culture (*n* = 4, three independent experiments) at day 7 from activation. Data are presented as the mean ± s.e.m. Statistical significance was assessed by a paired (**c**,**h**,**n**) or unpaired (**e**,**f**,**j**–**m**) two-tailed Student’s *t*-test. Source data

**Extended Data Fig. 6:**
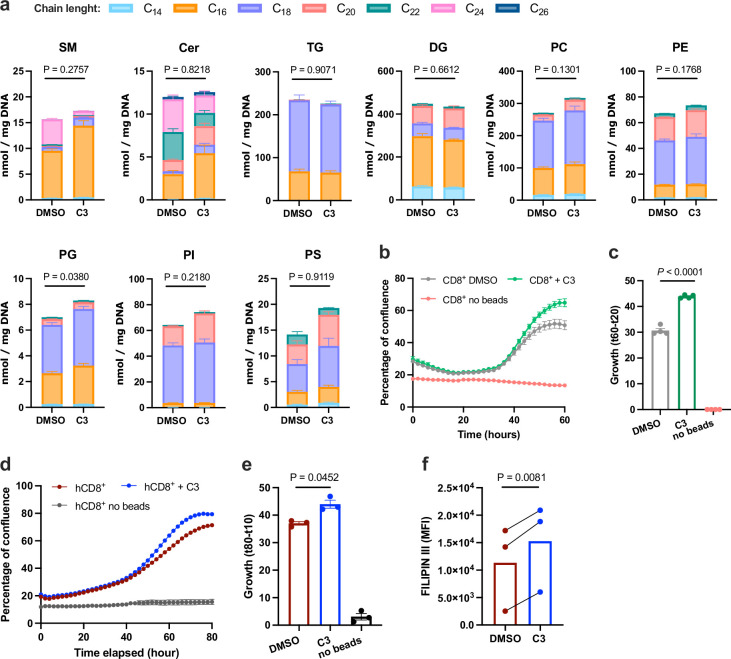
*Elovl1*-deficient CD8^+^ T cells present stronger TCR signalling and activation. **a**, Quantification of total lipid species by LS-MS standard lipidomics in in vitro-activated OT-I T cells treated with DMSO or C3 (n=5). For each lipid species, the contribution of fatty acids with different chain lengths is shown. The statistic is applied to the total quantity of lipids in each species. SM= sphingomyelins; Cer= Ceramides; TG= triacylglycerides; DG= diacylglycerides; PC= phosphatidylcholine; PE= phosphatidylethanolamine; PG= phosphatidylglycerol; PI= phosphatidylinositol; PS= phosphatidylserine. **b, c**, Representative real-time proliferation curves (**b**) and quantification of growth (% of confluence at t60h - % of confluence at t20h) (**c**) of naive murine CD8^+^ T cells stimulated with anti-CD3/anti-CD28 activation beads in the presence of ELOVL1 inhibitor (C3) in T cell media (n=4, two independent experiments). (**c**) Exact p value = 0.000006. **d, e**, Representative real-time proliferation curves (**d**) and quantification of growth (% of confluence at t80h- % of confluence at t10h) (**e**) of naive human CD8^+^ T cells stimulated with anti-CD3/anti-CD28 activation beads in the presence of ELOVL1 inhibitor (C3) (n=3, two independent experiments). **f**, Flow cytometry quantification of membrane cholesterol with FILIPIN III in ELOVL1 inhibitor (C3) and vehicle (DMSO) treated human CD8^+^ T cells at day 7 from in vitro activation (n=3). Data are presented as the mean ± s.e.m. Statistical significance was assessed by two-tailed unpaired (**a, c, e**) or paired (**f**) Student’s t-test. Source data

Elevated cholesterol in the plasma membrane has been reported to increase TCR clustering, leading to stronger TCR signalling and more potent antitumoural activity^38,39^. As upon *Elovl1* inactivation, we observed more membrane cholesterol and, both in vitro and in vivo RNA sequencing showed an upregulated T cell activation process compared with control CD8^+^ T cells (Figs. 5a and 6a,b), we next analysed this pathway. For this purpose, we took advantage of a known ELOVL1-specific inhibitor^43^, here referred to as C3. We first ran unbiased lipidomics on activated WT and C3-treated CD8^+^ T cells, which confirmed similar lipid modulations to the ones occurring upon *Elovl1* genetic deletion (Extended Data Fig. 6a). Next, we exploited direct stochastic optical reconstruction microscopy (dSTORM) to determine whether the lipid changes occurring upon ELOVL1 inhibition would impact TCR clustering and, as a consequence, its signalling. To do so we treated with C3 or /dimethylsulfoxide (DMSO) as control, naive CD8^+^ T cells for 6 h, when upregulation of free cholesterol was already detectable (Fig. 6c). Imaging of CD3, a component of the TCR complex, at single-molecule resolution, revealed that C3 treatment enhanced the clustering without affecting the total number of CD3 localizations at the plasma membrane (Fig. 6d–f). Moreover, activation with anti-CD3/anti-CD28 of naive CD8^+^ T cells pretreated 6 h with C3, resulted in more phosphorylation of LCK, ZAP70 and ERK1/2, the main mediators of TCR signalling cascade (Fig. 6g,h), and increased proliferation compared with nontreated CD8^+^ T cells (Fig. 6i–k and Extended Data Fig. 6b,c). Forty-eight hours after activation, when increased proliferation became evident, ELOVL1-inhibited CD8^+^ T cells still showed a significant increase of free cholesterol (Fig. 6l), along with a higher expression of the activation marker CD69 and the proliferation marker CD25 (Fig. 6m). Elevated free cholesterol and proliferation were also confirmed in naive CD8^+^ T cells isolated from human PBMCs, which were pretreated with C3 and activated with anti-CD3/anti-CD28, supporting the translatability of ELOVL1 targeting (Extended Data Fig. 6d–f). Stronger T cell activation was further validated in OT-I T cells genetically depleted for *Elovl1*. Upon 5 h of in vitro re-stimulation with KPC_OVA cancer cells, sg*Elovl1* OT-I T cells enhanced the release of cytotoxic granules compared with control OT-I T cells, as shown by higher exposure at the cell surface of CD107a, a surrogate marker of T cell degranulation and an indication of a stronger antigen recognition (Fig. 6n).

Altogether, these data show that ELOVL1 inhibition favours TCR clustering and signalling, leading to improved T cell activation and proliferation.

### *Elovl1* deletion rewires CD8^+^ T cells metabolism and sustains a memory phenotype

**Fig. 7:**
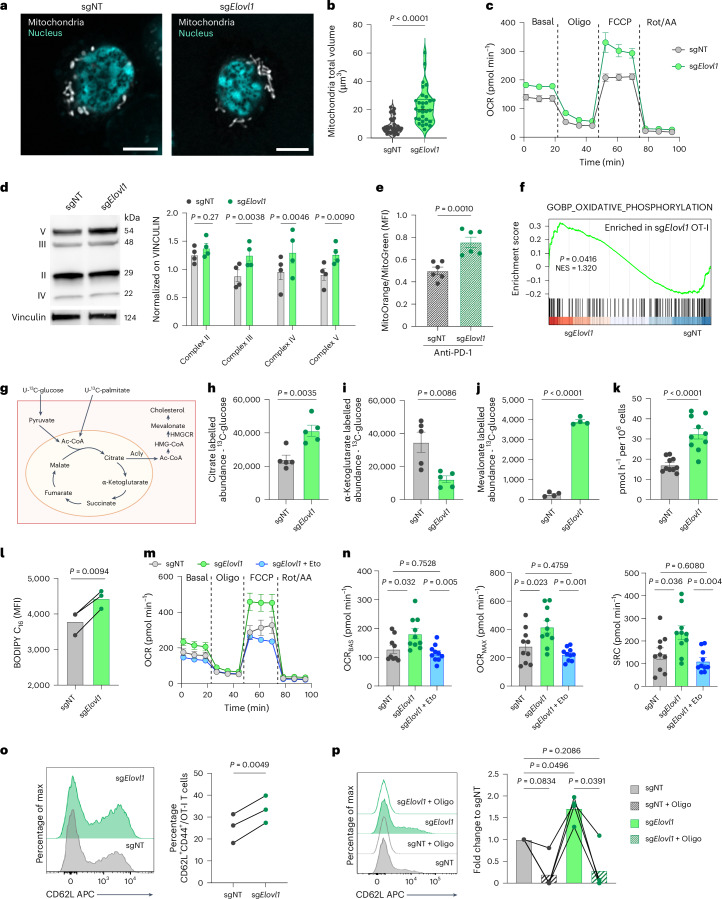
*Elovl1*-deficient CD8^+^ T cells increase mitochondrial functionality and are prone to memory differentiation. **a**,**b**, Representative images (scale bar, 5 µm, **a**) and mitochondria total volume quantification (**b**) in sgNT (*n* = 32) or sg*Elovl1* (*n* = 31) OT-I cells in vitro at day 7 (two independent experiments). **c**, Representative OCR of sgNT and sg*Elovl1* OT-I T cells in vitro at day 7 (*n* = 3, five technical replicates). **d**, Representative western blot and quantification of respiratory chain complexes in sgNT and sg*Elovl1* OT-I cells in vitro at day 7 (*n* = 4). **e**, Quantification of mitochondria function in sgNT and sg*Elovl1* OT-I cells infiltrating KPC_OVA primary tumour or mice treated with anti-PD-1. The ratio indicates mitochondrial potential normalized on mass (*n* = 6; two independent experiments). **f**, GSEA of oxidative phosphorylation evaluated via bulk-RNA sequencing of sgNT (*n* = 3) and sg*Elovl1* (*n* = 3) OT-I cells sorted, 7 days after ACT, from PDAC primary tumour of mice treated with anti-PD-1. **g**, Schematic of the ^13^C-glucose and ^13^C-palmitate tracing experiment with individual supplementation of each. **h**–**j**, Abundance of total ^13^C-labelled cellular citrate (**h**) α-ketoglutarate (**i**) and mevalonate (**j**) derived from ^13^C-glucose in sgNT or sg*Elovl1* OT-I T cells in vitro (*n* = 5). **k**, ^14C^C_2_O release from the conversion of [^14^C6] glucose-derived pyruvate to acetyl-CoA in sgNT and sg*Elovl1* OT-I cells in vitro at day 7 f (*n* = 5 two technical replicates). **l**, Flow cytometry quantification of Bodipy FL C_16_ uptake by sgNT and sg*Elovl1* OT-I cells (*n* = 3) at day 7. **m**,**n**, Representative OCR profile of sgNT, sg*Elovl1* or sg*Elovl1* OT-I pretreated for 1 h with 5 µM etomoxir in vitro at day 7 (**m**) and quantification of basal (OCR_BAS_), maximal (OCR_MAX_) and spare respiratory capacity (SRC) (**n**) (two independent experiments). **o**, Representative flow cytometry histogram and quantification of CD62L^+^ CD44^+^ (Tcm) in sgNT and sg*Elovl1* OT-I cells upon in vitro differentiation (*n* = 3, three independent experiments). **p**, Representative flow cytometry histogram and quantification of CD62L^+^CD44^+^ (Tcm) in sgNT and sg*Elovl1* OT-I cells, pretreated overnight with 1 µM oligomycin or DMSO, upon 48 h in vitro co-culture with KPC_OVA (*n* = 4, three independent exp). Data are presented as the mean ± s.e.m. Statistical significance was assessed by paired (**l**,**o**,**p**) or unpaired (**b**,**d**,**e**,**h**–**k**) two-tailed Student’s *t-*test and one-way ANOVA (**n**). Source data

**Extended Data Fig. 7:**
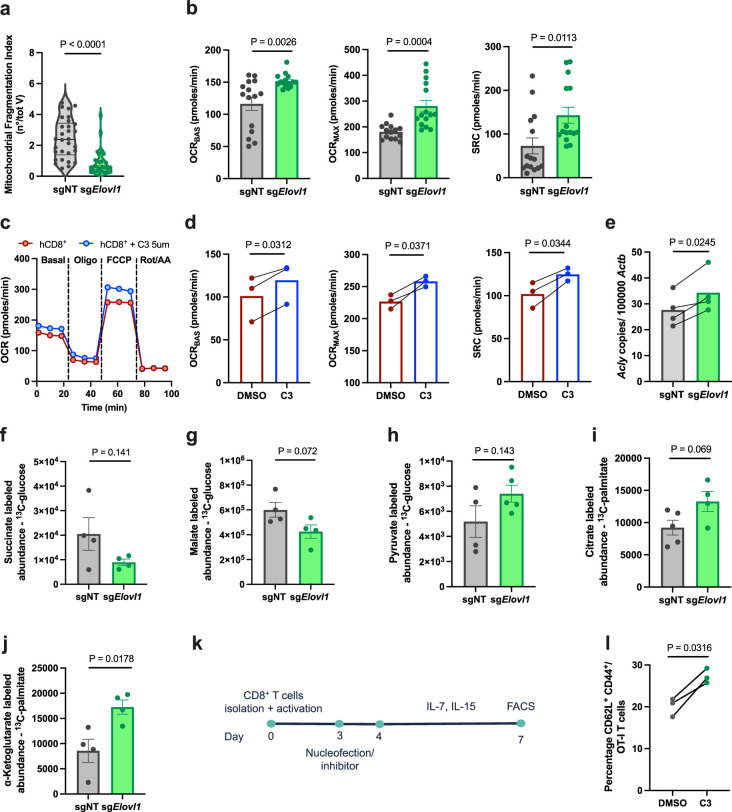
*Elovl1*-deficient CD8^+^ T cells increased mitochondrial functionality. **a**, Quantification of mitochondrial fragmentation via confocal microscopy of sgNT and sg*Elovl1* OT-I T cells at day 7 from in vitro activation (sgNT=30; sg*Elovl1*=30; three independent experiments). **b**, Quantification of Basal (OCR_BAS_), Maximal (OCR_MAX_), and Spare respiratory capacity (SRC), based on seahorse data of sgNT and sg*Elovl1* OT-I T cells 7 days after in vitro activation (n=3, five technical replicates). **c, d**, Representative oxygen consumption rate (OCR) profile of human CD8^+^ T cells treated with ELOVL1 inhibitor (C3) or vehicle (DMSO) at day 7 from in vitro activation (**c**) and quantification of Basal (OCR_BAS_), Maximal (OCR_MAX_), and Spare respiratory capacity (SRC), based on seahorse data (**d**) (n=3). **e**, ATP citrate lyase (*Acly*) expression assessed by qRT-PCR in vitro in sgNT or sg*Elovl1* OT-I T cells (n=4). **f**-**h**, Abundance of total ^13^C-labelled cellular Succinate (**f**), Malate (**g**), and Pyruvate (**h**) derived from ^13^C-Glucose (n=5). **i, j**, Abundance of total ^13^C-labelled cellular Citrate (**i**) and α-Ketoglutarate (**j**) derived from ^13^C-Palmitate (n=5). **k**, Schematics of in vitro differentiation memory assay. **l**, Quantification of central memory T cells (CD62L^+^ CD44^+^) in CD8^+^ T cells treated with C3 or control DMSO, upon in vitro differentiation with IL-7 and IL-15 (n=3). Data are presented as the mean ± s.e.m. Statistical significance was assessed by unpaired (**a, b, f-j**) or paired (**d, e, l**) two-tailed Student’s t-test. Source data

Mitochondria are crucial for high-energy-demand processes, including proliferation and antitumoural activity of cytotoxic T cells^44^, both of which are enhanced following *Elovl1* deletion. Therefore, we investigated mitochondrial morphology and function. In vitro, confocal imaging of activated sg*Elovl1* T cells showed more elongated mitochondria with significantly higher volume and lower fragmentation compared with sgNT CD8^+^ T cells (Fig. 7a,b and Extended Data Fig. 7a). Elongated mitochondria are often associated with better functionality^44,45^. Quantification of oxygen consumption rate (OCR) via Seahorse assay confirmed higher mitochondrial respiration in sg*Elovl1* compared with sgNT CD8^+^ T cells (Fig. 7c and Extended Data Fig. 7b). This was corroborated by significantly more respiratory chain complexes in *Elovl1*-deficient compared with control CD8^+^ T cells measured at protein (Fig. 7d) and RNA levels both in vitro and in vivo (Fig. 6b and Extended Data Fig. 5a). Similar results were obtained in in vitro-activated human CD8^+^ T cells treated with C3 or DMSO (Extended Data Fig. 7c,d). Higher mitochondrial functionality was also validated in vivo in sg*Elovl1* OT-I T cells infiltrating KPC_OVA primary tumour of mice treated with anti-PD-1 via MitoTracker staining and at the RNA level via gene set enrichment analysis (GSEA) of oxidative phosphorylation (Fig. 7e,f).

Considering the lipid and metabolic rewiring occurring upon *Elovl1* inhibition, we next exploited uniformly ^13^C-labelled glucose and palmitate (Fig. 7g) to fully characterize glucose and fatty acid metabolism, respectively. We observed that *Elovl1* deletion increased glucose incorporation into citrate (Fig. 7h). In the cytoplasm, citrate can be converted into acetyl-CoA by ATP citrate lyase (ACLY), providing a building block for cholesterol production. Accordingly, we observed higher *Acly* expression in sg*Elovl1* CD8^+^ T cells (Extended Data Fig. 7e). Additionally, reduced levels of glucose-derived tricarboxylic acid (TCA) intermediates α-ketoglutarate, succinate and malate (Fig. 7i and Extended Data Fig. 7f,g) were measured, along with an increased level of mevalonate (Fig. 7j), in line with the higher *Hmgcr* expression seen in sg*Elovl1* T cells (Fig. 5d). Consistent with higher citrate levels, we detected a slight increase in ^13^C-glucose-derived pyruvate (Extended Data Fig. 7h) and, by using radioactively labelled glucose, we confirmed that sg*Elovl1* CD8^+^ T cells convert more pyruvate to acetyl-CoA (Fig. 7k). Unlike with glucose, ^13^C-palmitate-derived citrate and α-ketoglutarate were both more abundant in sg*Elovl1* compared with control T cells (Extended Data Fig. 7i,j), suggesting that, in this step, palmitate-derived TCA intermediates are retained in the mitochondria. These findings were corroborated by the increased uptake of palmitate detected in sg*Elovl1* T cells (Fig. 7l) and by the ability of etomoxir, an inhibitor of carnitine palmitoyl transferase 1A (CPT1A), rate-limiting enzyme of palmitate oxidation, to normalize OXPHOS levels of sg*Elovl1* T cells to the ones of control cells (Fig. 7m,n). Altogether these findings prove that *Elovl1*-deficient CD8^+^ T cells oxidize glucose to sustain the production of cholesterol in the cytoplasm and use palmitate to replenish TCA cycle intermediates.

Considering that memory-like T cells rely on higher oxidative respiration and fatty acid uptake^46^, we then investigated whether *Elovl1*-deficient CD8^+^ T cells, due to their metabolism, were more prone to generate memory T cells. To this end, we genetically deleted *Elovl1* in in vitro-activated CD8^+^ T cells and cultured them only in the presence of IL-7 and IL-15, a cytokine combination driving memory phenotype (Extended Data Fig. 7k). Three days later, we observed that sg*Elovl1* CD8^+^ T cells had a higher percentage of central memory-like T cells (CD62L^+^CD44^+^) compared with sgNT T cells (Fig. 7o), in line with the in vivo observations. Similar results were obtained also when CD8^+^ T cells were treated with C3 (Extended Data Fig. 7l). Increased memory differentiation was further validated upon in vitro re-stimulation of OT-I T cells genetically depleted for *Elovl1*. After 2 days of co-culture with KPC_OVA cancer cells, sg*Elovl1* OT-I T cells presented a higher percentage of CD44^+^CD62L^+^ memory-like T cells, whereas inhibition of mitochondrial respiration through oligomycin pretreatment, completely abrogated their memory differentiation (Fig. 7p).

Together, these data indicate that *Elovl1* inhibition mediates a profound metabolic rewiring that primes T cells to differentiate more toward a memory-like phenotype.

## Discussion

T cell-based therapies, including T and CAR T cell transfer, have great therapeutic potential but are still confined in their use. To overcome this, CRISPR loss-of-function screens have been used to identify genes (metabolic and nonmetabolic), involved in memory/effector differentiation, driving T cell exhaustion and enhancing CAR T cell fitness in the TME^16,47,48^. These studies mostly focused on models of melanoma, breast cancer and glioblastoma, disregarding other cancer types and metastatic niches. Moreover, several therapies including immunotherapy are more effective against primary tumours than metastatic lesions, due to resistance mechanisms or the activation of alternative pathways^49,50^. Hereby, we exploited an orthotopic clinically relevant model of PDAC to perform an in vivo CD8^+^ T cell screening and collected, together with the primary tumour, also the metastatic sites (peritoneal metastasis, liver and lung) and lymphoid organs, aiming to uncover targets conferring an improved T cell persistence and fitness also in the metastatic niches.

ICB-based therapies have revolutionized cancer treatment. Among them, anti-PD-1 is used to reinvigorate T cell function in solid tumours, where constant antigen exposure and an unfavourable microenvironment induce T cell progression into exhausted states^48,51^. Notably, PD-1 blockade also rewires T cell metabolism, inducing glycolysis to sustain fast proliferation and differentiation into short-lived effector CD8^+^ T cells^18^. As T cell phenotypic states rely on distinct metabolic programmes, the metabolic pressure imposed by specific TMEs^52^ and concomitant therapies need to be considered when investigating ways to improve T cell fitness^53^. For these reasons, we performed the in vivo screens in the presence of anti-PD-1 therapy to identify possible synergism and achieve greater results.

In vivo CD8^+^ T cell CRISPR screens, typically quantify T cell infiltration, which is not always correlated with improved functionality. This highlights the need for adjunct functional studies exploiting other omics^54,55^. Based on the above criteria, our initial multi-organ in vivo CRISPR screen identified 83 significantly enriched genes that underwent a refinement screening. The CRISPR technology was combined with scRNA-seq (CROP-seq) to unveil the transcriptomic profile, and therefore the phenotypes, acquired in vivo upon each T cell metabolic alteration. This high-throughput technique allowed us to select targets for their ability to sustain T cell proliferation, effector function and memory differentiation, while counteracting exhaustion.

These platforms highlighted *Elovl1* as a particularly appealing target to favour proliferation, effector and memory functions in synergy with anti-PD-1 treatment. Of note, *Elovl1*, which was enriched in the liver in the initial screening, proved to be a highly efficient target to combat primary tumour and peritoneal metastasis, supporting the relevance of our multi-dimensional approach in identifying promising targets with enhanced systemic fitness. Collectively, our single-cell screening platform offers the opportunity to discover targets promoting specific CD8^+^ T cell phenotypes.

**Extended Data Fig. 8:**
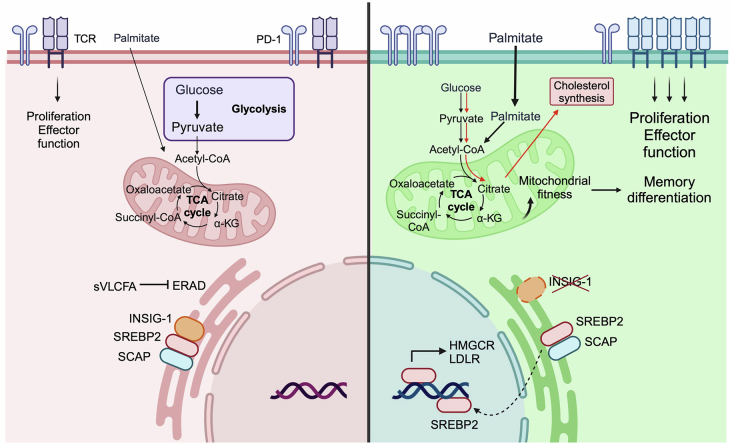
Scheme illustrating the role of Elovl1 in CD8^+^ T cells. Upon a multi-organ metabolic CD8^+^ T cell CRISPR/Cas9 screening in vivo followed by a scRNA-seq screening in the context of PDAC, *Elovl1* was identified as a promising metabolic target to sustain proliferation and both effector and memory phenotypes in CD8^+^ T cells. Accordingly, ACT of *Elovl1*-deficient CD8^+^ T cells in combination with anti-PD-1 promoted tumour reduction in preclinical models of PDAC and melanoma, and in patients, low expression of *ELOVL1* in CD8^+^ T cells correlated with anti-PD-1 response. Mechanistically, *Elovl1* inactivation results in the reduction of saturated VLCFA incorporated in many lipid species among which sphingomyelins and ceramides. This mediates the destabilization of INSIG1, consequent SREBP2 activation and cholesterol accumulation. Cholesterol synthesis is supported by increased glucose oxidation while palmitate fuels oxidative phosphorylation. The rewired lipid composition enhances the formation of membrane microdomains, leading to improved TCR clustering and signalling with sustained effector functions and proliferation. Complementarily, mitochondrial reprogramming mediates memory differentiation. On the contrary, *Elovl1* WT T cells present more fragmented mitochondria and rely on glycolysis to sustain their proliferation, which limits their function in a tumour microenvironment deprived of glucose. Altogether, *Elovl1* targeting synergizes with anti-PD-1 to promote effective T cell responses. Created in BioRender. https://BioRender.com/t10b575.

*Elovl1* has been widely studied in brain diseases such as adrenoleukodystrophy and certain tumour types as an unfavourable prognostic marker^56–58^. However, its role in CD8^+^ T cells in the context of cancer or other diseases is unknown. We showed that CD8^+^ T cells enhanced *Elovl1* expression during proliferation, whereas naive and differentiated cells downregulate it. The need of proliferating cells for lipids to sustain membrane formation during division could explain the increase in *Elovl1* expression. However, the observation that *Elovl1* inhibition hampers T cell proliferation, suggests that *Elovl1* might work as a metabolic checkpoint to control excessive T cell activation and function. Here, we confirmed that *Elovl1* targeting decreases saturated/monounsaturated VLCFAs incorporated in many lipid species, including sphingomyelins and ceramides. Previous studies in yeast have shown that INSIG1 can be anchored in the ER membrane due to the accumulation of saturated VLCFAs^42^, while in mammals, polyunsaturated fatty acids (PUFAs) have been shown to stabilize ubiquitinated INSIG1, delaying its degradation^59^. Here, we established a connection between saturated VLCFAs and the stabilization of INSIG1. Accordingly, *Elovl1*-deficient CD8^+^ T cells exhibit increased activation of SREBP2, which boosts both cholesterol synthesis (through HMGCR) and uptake (via LDLR), ultimately leading to the accumulation of free cholesterol (Extended Data Fig. 8). Sphingomyelins and ceramides, together with cholesterol, are the main components of the plasma membrane and have been reported to interact and form membrane microdomains^60,61^. Increments of free cholesterol in the plasma membrane have been shown to mediate higher T cell activation and proliferation, by favouring TCR clustering in microdomains^62^. Alongside, also LDLR expression in the membrane of CD8^+^ T cells plays a pivotal role by sustaining TCR recycling and signalling^63^. Therefore, ways to augment free cholesterol accumulation such as inhibition of cholesterol esterase (ACAT1) or sustaining LDLR activity by blocking tumour cell-derived proprotein convertase subtilisin/kexin type 9 (PCSK9), proved to be effective in potentiating CD8^+^ T cell activity in tumours^38,39,63^. In line with this, we observed that in vitro pharmacological inhibition of ELOVL1 in naive CD8^+^ T cells augmented free cholesterol pool accumulation at the plasma membrane, which favoured TCR complex clustering and signalling, leading to enhanced activation and proliferation. This was accompanied by increased expression of activation markers such as CD69 and CD25. In vivo *Elovl1*-deficient CD8^+^ T cells, due to their stronger activation, expressed higher levels of co-inhibitory modules including PD-1, TIGIT and TIM-3. Although known for their role in CD8^+^ T cell response contraction^64,65^, more studies are showing that co-inhibitory molecules can also reflect their activation and effector states, if accompanied by a sustained cytotoxic functionality^66^. Additionally, TIM-3 can also identify Tpex transitioning to an effector state^67^. Considering that solid tumours are characterized by a high infiltration of immunosuppressive cells expressing checkpoint molecules such as PD-L1/PD-L2 (refs. ^68–70^), we showed that *Elovl1*-deficient CD8^+^ T cells particularly benefit from anti-PD-1 treatment and synergize with it to unleash their potentiated antitumoural activity, thus mediating tumour reduction.

Nutrient deprivation and toxic metabolites in the TME can reprogramme CD8^+^ T cell metabolism and induce their exhaustion. Lipids, among other nutrients, are essential membrane components and signal transducers that can promote or inhibit T cell functions. During tumour progression, long-chain fatty acids and VLCFAs are often accumulated in the TME, along with glucose deficiency.

In this microenvironment, T cells need a switch to fatty acid oxidation (FAO), consisting in the catabolism of long-chain fatty acids to sustain their energy requirements. Previous studies showed that enhancing FAO by engaging PPAR-α^71^ or by restricting glycolysis^72^ can augment T cell antitumoural activity.

On the contrary accumulation of long-chain fatty acids or sustained fatty acid synthesis through acetyl-CoA carboxylase can impede FAO, thus leading to T cells dysfunction^73,74^. Moreover, uptake and accumulation of VLCFAs and cholesterol in intracellular LDs, is deleterious for CD8^+^ T cells, leading to enhanced lipid peroxidation and ferroptosis^73,75,76^.

Here we show that the deletion of *Elovl1* potentiates mitochondrial OXPHOS as a source of energy. Accordingly, these cells increase their palmitate uptake and utilization, while avoiding accumulation as suggested by similar triglycerides and LD quantification. On the other hand, they shift glucose usage to sustain cholesterol synthesis.

ACT approaches, including CAR T cell therapy, showed poor efficacy in solid tumours due to their limited persistence and fast differentiation into dysfunctional and exhausted states. It has been proposed that the persistence of infused T cells is higher when cells retain memory-like phenotypes and can sustain proliferation in the harsh TME^47,77–80^. With our approach, we unveil a metabolic target that, by synergizing with anti-PD-1, potentiates CD8^+^ T cell proliferation and effector functions while retaining a memory phenotype, crucial for their long-term maintenance. Considering that we manipulated antigen-specific T cells (OT-I and Pmel-1), genetic deletion of *Elovl1* could be applied to CAR T directed against solid tumour antigens, to sustain their persistence and at the same time sensitize the tumour to ICB treatment.

In conclusion, by employing a functional metabolic survey in vivo, we identified *Elovl1* as a metabolic checkpoint in CD8^+^ T cells. We demonstrated that manipulation of *Elovl1* in CD8^+^ T cells increases the incorporation of saturated long-chain fatty acids (C_14_–C_18_) in most of the lipid species including sphingomyelins and ceramides, and favours INSIG1 degradation, thus inducing SREBP2 activation. Augmented cholesterol levels lead to stronger TCR signalling and proliferation. Alongside a profound lipid rewiring *Elovl1* deficiency promotes FAO and mitochondrial fitness, which sustain memory differentiation. in vivo*, Elovl1* deletion and ICB therapy synergize to overcome immunotherapy resistance in PDAC and melanoma preclinical models (Extended Data Fig. 8).

### Limitations of study

In this work, we identified ELOVL1 as a metabolic checkpoint promoting the reprogramming of cellular metabolism, and leading to increased fitness and tumour control in synergy with anti-PD-1.

For feasibility reasons, the initial screening identifying *Elovl1* was conducted by introducing ovalbumin in the cancer cells, which is a non-endogenous antigen. Moreover, only genetic ablation of *Elovl1* was tested for ACT, whereas the long-term effect of pharmacologically inhibited CD8^+^ T cells was not explored.

We provided dSTORM imaging of CD3 clustering in naive CD8^+^ T cells upon ELOVL1 pharmacological inhibition. However, to dissect how *Elovl1* loss alters microdomain functions and TCR signalling, considerable mechanistic studies will be required, involving further single-molecule imaging and lipidomics on isolated plasma membranes.

Our study focuses on the role of ELOVL1 in the CD8^+^ T cell compartment. Further work is required to investigate the role of ELOVL1 in other cell populations. This might give more indications on the possible use of systemic pharmacological ELOVL1 inhibition, as a promising therapeutic strategy.

Finally, given the paucity of available biopsies from patients with PDAC under anti-PD-1 treatment, we could not validate the role of ELOVL1 in human PDAC cohorts and therefore relied on a dataset of patients with melanoma, where anti-PD-1 is a first-line treatment.

## Methods

### Experimental model and study participants details

#### Cell lines

The KPC pancreatic cell line used in this study (FC1245) was kindly provided by the laboratory of D. A. Tuveson and was derived from spontaneous tumours arising in the KPC (Kras^LSL.G12D/+^; p53^R172H/+^; Pdx: CreTg^/+^) pancreatic cancer mouse model. An OVA-expressing KPC cell line was established by stable transduction of the parental cell line with a lentiviral vector harbouring the expression cassette of the ovalbumin (OVA)_257–264_ immunogenic ‘SIINFEKL’ peptide and was maintained in DMEM (Thermo Fisher) with 10% FBS (Gibco), 1% penicillin–streptomycin (pen/strep; Gibco), 1% sodium pyruvate (Gibco) and geneticin (G418, InvivoGen).

The B16F1 melanoma cell line was originally obtained from the American Type Culture Collection (ATCC) (CRL-6323) and maintained in complete RPMI 1640 + Glutamax medium (Thermo Fisher) containing 10% FBS (Gibco), 100 IU ml^−1^ penicillin and 100 μg ml^−1^ streptomycin (Gibco). HEK293 cells were obtained from ATCC (CRL-1573) and cultured in DMEM supplemented with 10% FBS, 100 U ml^−1^ pen/strep and 2 mmol l^−1^ glutamine (Gibco). Cells were cultured at 37 °C and 5% CO_2_. All the cell lines were passaged in the laboratory for no longer than ten passages after receipt and tested for *Mycoplasma* by PlasmoTest-Mycoplasma Detection kit (InvivoGen) every 6 months.

#### Primary cell culture

Mouse T cells were freshly isolated from spleens of both male and female mice between 6 and 10 weeks of age.

For human studies, T cells were isolated from buffy coats of healthy male and female volunteers aged between 25 and 65 years provided by Red Cross Donor Center Mechelen, Belgium (institutional approval S68611) (anonymized). Donors provided written consent.

#### Mice

OT-I:Rosa26-Cas9 mice (C57BL/6J background) were generated by intercrossing Rag2/OT-I mice with Rosa26-Cas9 knock-in mice, which constitutively express the Cas9 nuclease. OT-I mice express an αβ TCR recognizing ovalbumin peptide residues 257–264 (OVA_257–264_) in the context of H2-K^b^.

Rosa26-Cas9 immunocompetent mice (C57BL/6J background) and C57BL/6J wild type (WT) were used as recipient mice and were inoculated with KPC_OVA, KPC or B16F1 cells.

Pmel-1 mice (C57BL/6J background) express an αβ TCR recognizing human and mouse gp100_25–33_ epitope presented on H2-D^b^. All mice used were between 6–12 weeks old, without specific sex selection. In all experiments, mice were randomly assigned to the different experimental groups, to have a similar weight average and s.d. Mice were maintained under pathogen-free, temperature- and humidity-controlled conditions under a 12-h light–dark cycle and received normal chow (ssniff R/M-H). A humane end point was reached with 20% of body weight loss or 1,500 mm^3^ tumour size. The maximal tumour burden was never exceeded. Killing was performed by cervical dislocation or CO_2_. Housing conditions and all experimental animal procedures were approved by the Animal Ethics Committee of the KU Leuven (P226/2017) and the Landesverwaltungsamt and LANUV (81-02.04.2020.A355), NRW, Germany. The phenotypes were observed indiscriminately in male and female mice. No sex-related issues applied to this work.

### Method details

#### CD8^+^ T cell isolation and culture

For lentiviral transduction CD8^+^ T cells were prepared as follows: spleen and lymph nodes (four superficial cervicals, two axillary and two branchial, two inguinal and two lumbar) were isolated from OT-I:Rosa26-Cas9 mice. The organs were then mechanically dissociated in a 70-μm cell strainer. Red blood cells were lysed in Red Blood Cell Lysing Buffer (Sigma-Aldrich), incubated for 2 min at 37 °C, washed and filtered through a 40-μm cell strainer. CD8^+^ T cells were isolated using the mouse CD8^+^ T cells Isolation kit (MojoSort) according to the manufacturer’s guidelines. Isolated T cells were cultured for 24 h in T cell medium (RPMI 1640 (Thermo Fisher), 10% of FBS (Gibco), 1% pen/strep (Gibco), 0.1% 2-mercaptoethanol (Gibco), 1% Non-Essential Amino Acids Solution (Gibco) and 1% sodium pyruvate (Gibco)) with a 1:1 ratio of Mouse T-Activator CD3/CD28 Dynabeads (Thermo Fisher Scientific). For the following 2 days, activated CD8^+^ T cells were expanded in T cell medium supplemented only with 10 ng ml^−1^ mIL-2, 5 ng ml^−1^ mIL-7 and 5 ng ml^−1^ mIL-15 (all from PeproTech), and used for lentiviral transduction.

For nucleofection OT-I T cells were prepared as follows: spleen and lymph nodes were isolated from OT-I mice and processed as described above. Total splenocytes and lymphocytes were resuspended in T cell medium added with 1 μg ml^−1^ OVA257–264 peptide in the presence of 10 ng ml^−1^ mIL-2, 5 ng ml^−1^ mIL-7 and 5 ng ml^−1^ mIL-15 for 3 days, then used for nucleofection. For nucleofection, Pmel-1 T cells were isolated from spleens collected from Pmel-1 TCR-transgenic mice as described above, resuspended in T cell medium supplemented with IL-2, seeded at 10^6^ per ml onto an anti-CD3/CD28 (BioLegend; 1 μg ml^−1^) coated six-well plate for 3 days and then used for nucleofection.

#### CD8^+^ T cell lentiviral transduction

CD8^+^ T cells were transduced with the vector of choice on day 2 post-isolation, by adding to the medium 10 µg ml^−1^ protamine sulfate (Sigma-Aldrich), 10 ng ml^−1^ mIL-2, 5 ng ml^−1^ mIL-7 and 5 ng ml^−1^ mIL-15 and the proper volume of concentrated lentivirus to have a multiplicity of infection of 80–100. The cells were then expanded for 5 days in T cell medium supplemented with 10 ng ml^−1^ mIL-2, 5 ng ml^−1^ mIL-7 and 5 ng ml^−1^ mIL-15 to provide time for sgRNA expression. Transduction with the libraries was performed on a total of >2 × 10^7^ Cas9 OT-I T cells to achieve an initial library coverage of >2,000×.

#### CD90.1^+^ OT-I T cell isolation

Transduced CD90.1^+^ OT-I T cells were selected via magnetic positive selection using CD90.1 MicroBeads (Miltenyi Biotech) and LS magnetic columns (Miltenyi Biotech), according to the manufacturer’s instructions. CD90.1 isolation was performed 4 days post-transduction, immediately before ACT. An aliquot of the cells was taken before and after the isolation for FACS analysis to determine the efficiency of transduction and the purity of the isolated CD90.1^+^ OT-I T cell population.

#### CD8^+^ T cell nucleofection

The validation of the screening was performed with the use of electroporation^81^. Splenocytes and lymphocytes were isolated, activated and cultured as described above. The nucleofection was performed 3 days after OT-I T or Pmel-1 T cells isolation. For this purpose, the Alt-R CRISPR-Cas9 RNA (Alt-R crRNA, IDT) of choice and the Alt-R trans-activating crRNA (Alt-R tracRNA, IDT) were mixed in equimolar concentrations to have a final duplex concentration of 50 µM and the annealing was performed as follows: 95 °C 5 min; 90 °C 2 min; 85 °C 2 min; 80 °C 2 min; 75 °C 2 min; 70 °C 2 min; 65 °C 2 min; 60 °C 2 min; 55 °C 2 min; 50 °C 2 min; 45 °C 2 min; 40 °C 2 min; 35 °C 2 min; 30 °C 2 min; 25 °C inf. RNP complexes were then generated by incubating duplex RNA with the Cas9 enzyme in a 3:1 ratio at room temperature for 20 min. OT-I or Pmel-1 T cells were collected, washed twice in PBS and resuspended at a concentration of 1 × 10^8^ per ml in P4 Nucleofector solution (P4 Primary Cell 4D-Nucleofector X kit L, Lonza). Then, 1 × 10^7^ OT-I or Pmel-1 T cells were incubated with the RNP complex at /room temperature for 2 min, transferred to the cuvette (P4 Primary Cell 4D-Nucleofector X kit L, Lonza) and electroporated with the programme CM137 on a 4D-Nucleofector System (Lonza). The cells were then collected and maintained in culture at a concentration of 0.5–2 × 10^6 ^T cell medium added with 10 ng ml^−1^ mIL-2, 5 ng ml^−1^ mIL-7 and 5 ng ml^−1^ mIL-15 for the next 3 days, when they were used for ACT or in vitro assays.

#### In vivo screenings

KPC_OVA murine cells were detached with 0.25% Trypsin-EDTA (Gibco), collected in PBS (Gibco) and counted. Then, 1 × 10^6^ KPC_OVA cells were resuspended in 20 µl and injected orthotopically in the pancreas head of recipient Rosa26-Cas9 mice. ACT was performed with 2 × 10^6^ CD90.1^+^ OT-I T cells 5 days after KPC_OVA injection. For the in vivo metabolic screen, an aliquot of CD90.1^+^ T cells was pelleted and frozen at −20 °C for next-generation sequencing (NGS) analysis (T0). Mice were killed 7 days after ACT and spleen, draining and nondraining lymph nodes, primary tumour, liver, lungs and peritoneal metastasis were collected and processed for sorting of OT-I T cells. Samples collected were sequenced in three independent runs and pooled in the following analysis. For the CROP-seq, only the primary tumour was collected and processed for CD90.1^+^ OT-I T cells sorting.

#### Tumour model for target validation

For the PDAC mouse model, KPC_OVA were detached as previously described. Then, 4 × 10^4^ cells were resuspended in 20 µl PBS and injected orthotopically in the pancreas head of recipient mice. ACT of 5 × 10^6^ engineered OT-I T cells was performed 7 days after KPC_OVA injection. For the experimental liver metastasis model, 1 × 10^5^ KPC_OVA were injected intrasplenic, followed by splenectomy. ACT of 5 × 10^6^ engineered OT-I T cells was performed 8 days after KPC_OVA injection. For both models, mice were killed 7 days post-ACT and relevant organs were collected and processed for FACS (sorting or analysis) or histological analysis. For the melanoma mouse model, 2 × 10^5^ B16F1 melanoma cells were injected in 100 μl PBS into the flank of C57BL/6 mice. ACT with engineered pmel-1 T cells was performed as described previously^82^. In brief, when B16F1 tumours reached a size of 10–25 mm^2^, mice received a single dose of cyclophosphamide intraperitoneally (i.p.) (100 mg kg^−1^ body weight in 100 μl PBS; d−1) followed the next day by intravenous injection of 2 × 10^6^ engineered CD90.1^+^CD8^+^ pmel-1 T cells and 5 × 10^8^ plaque-forming units of a recombinant Ad-gp100 i.p. (d0). Then, 50 μg CpG 1826 (Invivogen) and 50 μg polyinosinic:polycytidylic acid (poly(I:C), Invivogen) in 100 μl distilled water were injected peritumourally 3, 6 and 9 days after adoptive pmel-1 T cell transfer. Tumour size was measured 2–3 times weekly. The tumour area was calculated in mm^2^ using the equation: *A* = length × width. Mice with tumours reaching 100 mm^2^ were killed.

#### ICB treatment

Anti-PD-1 (BioLegend) and the control immunoglobulin G (IgG) from rat serum (Sigma-Aldrich) were administered at a dosage of 10 mg kg^−1^, through i.p. injection. Anti-PD-1 and IgG were diluted in PBS. The treatment was given from the day of ACT every 2 days in the screenings and from the day after ACT every 2 days for the target validation experiments. In the ACT experiments using pmel-1 T cells, 10 mg kg^−1^ of anti-PD-1 was injected i.p. in 100 μl PBS on day 3, 6 and 9 after T cell transfer.

#### Flow cytometry

Tumours were collected, weighed and kept in ice-cold PBS. The tumour mass was then mechanically dissociated in digestion buffer (minimum essential medium-α (αMEM; Lonza) supplemented with 1% pen/strep, 50 μΜ β-mercaptoethanol, 5% FBS, 5 U ml^−1^ DNase I (Sigma-Aldrich), 0.85 mg ml^−1^ Collagenase V *(*Sigma-Aldrich*)*, 1.25 mg ml^−1^ Collagenase D (Sigma-Aldrich) and 1 mg ml^−1^ Dispase (Gibco). Tumour pieces were collected into gentleMACS C tubes (Miltenyi Biotec) and dissociated by using first the h_cord_1 programme of an automatic tissue gentleMACS Dissociator (Miltenyi Biotec) and then incubated for 40 min at 37 °C.

Peritoneal metastases were collected and mechanically dissociated in 5 ml of the same digestion buffer used for the primary tumour. The pieces were then collected into gentleMACS C tubes (Miltenyi Biotec) and dissociated by using the 37C_m_TDK_1 programme.

Lungs and livers were collected and dissociated with 10 ml of lung and liver Digestion Buffer (RPMI supplemented with 1% pen/strep, 5% FBS, 40 U ml^−1^ DNase I (Sigma-Aldrich), 1 mg ml^−1^ Collagenase I (Sigma-Aldrich) and 2 mg ml^−1^ Dispase (Gibco)) in C tubes (Miltenyi Biotec) using the 37C_m_LDK_1 programme. The digestion was then stopped with FACS buffer and the sample was filtered through a 70-µm cell strainer. Red blood cell lysis was performed by using Hybri-Max (Sigma-Aldrich, R7757). The sample was then passed through a 40-µm cell strainer to result in a single-cell suspension. Spleens were recovered from mice and weighed. The dissociation into a single-cell suspension was performed as previously described. Single cells were resuspended in FACS buffer (PBS containing 2% FBS and 2 mmol l^−1^ EDTA) and incubated for 15 min with Mouse BD Fc Block purified anti-mouse CD16/CD32 (1:25 dilution, BD Pharmingen). Extracellular staining was performed for 30 min at 4 °C. For the intracellular measurement of IFNγ, TNF, IL-2 and GZMB, single-cell suspensions were resuspended in RPMI (10% FBS and 1% pen/strep) and stimulated with phorbol 12-myristate 13-acetate/ionomycin cell stimulation cocktail (Invitrogen, 1:500 dilution) in the presence of brefeldin A (BioLegend; 1:1,000 dilution) and monensin (Invitrogen; 1:1,000 dilution) for 4 h (37 °C). The cells were then washed in FACS buffer and stained with a viability dye and extracellular markers. To perform intracellular staining, cells were permeabilized by using the Foxp3/Transcription Factor Fixation/Permeabilization kit (Invitrogen) according to the manufacturer’s instructions and incubated overnight at 4 °C with the specific intracellular antibodies. For the SREBP2 nuclear translocation analysis, purified rabbit anti-mouse SREBP2 (1:200 dilution) was incubated overnight at 4 °C in Permeabilization buffer (Invitrogen), then the cells were washed and incubated for 1 h with a donkey anti-rabbit-A488 and 7-AAD, for DNA staining. For MitoTracker staining (Thermo Fisher Scientific), 500,000 cells were incubated with 50 nM MitoTracker green and 25 nM MitoTracker Orange for 30 min at (37 °C), followed by intracellular staining as described above. Cells were subsequently washed and resuspended in FACS buffer before flow cytometric analysis by a FACS Canto II, Fortessa X-20 or flow sorting by a FACS Aria III, Aria Fusion (BD Biosciences). For the SREBP2 nuclear translocation, the cells were acquired on a BD FACSDiscover S8 Cell Sorter with BD CellView. Data were acquired by FACSDiva (v.9.0) and FACSChorus (v.5.1) software and analysed by FlowJo (TreeStar, v.10.9). Fluorescence minus one controls were utilized to ensure proper gating of positive populations.

#### Metabolic libraries design and cloning

The library used for the in vivo metabolic screening on CD8^+^ T cells was synthetized as previously described^22^.

Similarly, the library used for in vivo CROP-seq, made of 246 sgRNA targeting the 83 distilled candidate genes and 34 nontargeting sequences was synthesized and cloned into CROP-seq-Guide-Thy1.1.

#### Next-generation sequencing on gDNA

Genomic DNA (gDNA) was isolated using DNeasy blood and tissue kit (QIAGEN) following the manufacturer’s guidelines. PCR of gDNA was performed to attach sequencing adaptors and barcode samples. For each sample, the gDNA was split into multiple 25-μl PCR reactions (total volume) containing a maximum of 1 μg gDNA. PCR mixture per reaction: 12.5 μl KAPA HIFI HOT START MIX (2×), 1 μl P5 stagger primer mix (stock at 10 μM concentration), 1 μl of a uniquely barcoded P7 primer (stock at 10 μM concentration), adding mQ water and gDNA input (max 1 μg per reaction) to 25 μl. PCR cycling conditions: an initial 2 min at 98 °C; followed by 30 s at 98 °C, 30 s at 60 °C, 30 s at 72 °C, for five cycles + additional 20–25 cycles of 30 s at 98 °C, 30 s at 65 °C, 30 s at 72 °C and a final 5 min extension at 72 °C. P5 and P7 primers were synthesized at IDT. Next, PCR products were purified with Agencourt AMPure XP SPRI beads according to the manufacturer’s instructions (Beckman Coulter). DNA concentrations were measured, and samples were equimolarly pooled and subjected to Illumina NGS. Mapped read counts were subsequently used as input for the MAGeCK analysis software package^23^ and STARS^31^.

#### scRNA-seq (CROP-seq)

Single-cell libraries were prepared using the Chromium Next GEM Single Cell 3′ _v.3.1 kit (10x Genomics). In brief, the single-cell suspensions were loaded onto the Chromium Controller according to their respective cell counts to generate 9,000 single-cell gel beads in emulsion (GEMs) per sample. Each sample was loaded into a separate channel. The complementary DNA content of each sample after complementary DNA amplification of 11 cycles was quantified and quality checked using a high-sensitivity DNA chip in a tapestation (Agilent). Then, 25% of cDNA from the previous step was used for fragmentation, end repair and A-tailing, followed by adaptor ligation and PCR indexing. After library quantification and quality checking by tapestation (Agilent), samples were diluted and loaded onto the NovaSeq (Illumina) to a sequencing depth of 500 million reads per sample (approximately 50,000 reads per cell).

#### Bulk-RNA sequencing

In vitro, OT-I T cells transduced with a lentiviral vector harbouring a nontargeting or *Elovl1* single guide RNA were sorted for CD90.1 expression at day 7 from activation (3 days after transduction). Total RNA was isolated using the RNeasy Mini kit (QIAGEN) and resuspended in RNase-free water. Frozen RNA was shipped to Novogene for the Plant and Animal Eukaryotic Strand Specific mRNA (WOBI) service. The resulting 150-bp reads were sequenced paired-end on an Illumina Novaseq 6000 instrument.

In vivo, OT-I T cells transduced with a lentiviral vector harbouring a nontargeting or *Elovl1* single guide RNA were sorted for CD90.1 expression, 7 days post-ACT, from PDAC primary tumour of mice treated with anti-PD-1. Cells from different mice were kept separately and considered as a biological replicate. Total RNA was extracted using TRIzol (Thermo Scientific) according to the manufacturer’s guidelines and resuspended in RNase-free water. Frozen RNA was shipped to Novogene and pre-amplified with the SMARter amplification kit, followed by Plant and Animal Eukaryotic Strand Specific mRNA (WOBI) service. The resulting 150-bp reads were sequenced paired-end on an Illumina Novaseq X Plus instrument.

#### RNA extraction, reverse transcription and RT–qPCR

RNA was extracted using the RNeasy Mini kit (QIAGEN) according to the manufacturer’s instructions. Reverse transcription was performed with the Superscript IV First Strand cDNA Synthesis kit (LifeTechnologies) according to the manufacturer’s instructions. cDNA, primers and Power Up SYBR Green Master Mix (Applied Biosystems) were prepared in a volume of 20 μl. Samples were loaded into an optical 96-well Fast Thermal Cycling Plate and quantitative PCR with reverse transcription (RT–qPCR) was performed using an ABI Prism 7500 Real-Time PCR. Data were normalized to housekeeping gene expression glyceraldehyde 3-phosphate dehydrogenase (GAPDH) or Actin β. Primer sequences are given in Supplementary Table 1.

#### Radiolabeling assay

For glucose oxidation, 1 × 10^6 ^T cells were incubated in T cell medium containing 0.1 μCi μl^–1^ [6-14 C]-d-glucose (PerkinElmer) for 6 h. Cells were then lysed with 12% perchloric acid and radioactive CO_2_ was captured on Whatman paper soaked in hyamine overnight at room temperature. Radioactivity was determined by liquid scintillation counting.

#### Seahorse metabolic assay

OCR was measured following the manufacturer’s instructions of Seahorse XF Cell Mito Stress Test kit (Agilent). In brief, 2 × 10^5 ^T cells (mouse or human) were resuspended in nonbuffered DMEM (Sigma-Aldrich, D5030; pH 7.4, 10 mM glucose and 2 mM glutamine) and then plated in a poly-d-lysine-coated XF96 plate. OCR was measured using an XF96 Extracellular Flux Analyzer (Seahorse Bioscience) upon sequential treatment with 1 µM oligomycin, 2 µM FCCP and 0.5 µM rotenone/antimycin A. Data were normalized to cell number.

#### ^13^C labelling, liquid chromatography–mass spectrometry

##### Metabolite extraction

After a 6 h incubation period of ^13^C-glucose and ^13^C-palmitate, cells were washed with room-temperature saline and quenched by the addition of cold methanol. Subsequently, methanol:water:chloroform extraction (5:3:5 ratio) was added to the samples and the mixture vortexed for 10 min at 4 °C followed by centrifuging at 16,000*g* for 10 min to achieve phase separation. The methanol–water phase, which contains polar metabolites, was separated and dried using a SpeedVac for 24 h. The remaining protein from the extraction was dissolved in 0.2 mM KOH overnight and then quantified using the Pierce BCA Protein Assay kit (Thermo Scientific).

##### Mass spectrometry

Dried metabolite extracts were resuspended in HPLC-grade water containing 1 μM piperazine-*N*, *N*′-bis (PIPES) added as an internal standard and transferred to HPLC vials for analysis. For 3-NPH derivatization, 5 μl of resuspended metabolites was mixed with 5 μl of 250 mM 3-NPH in 75% methanol aqueous solution, 5 μl of 150 mM EDC in methanol and 5 μl of 7.5% pyridine in methanol. Next, samples were incubated at 4 °C for 2 h and 4 μl of 9 mM BHT in methanol was added followed by 56 μl of HPLC water. Subsequently, samples were centrifuged at 15,000*g* for 5 min at 4 °C and transferred to HPLC vials for analysis. Liquid chromatography–tandem mass spectrometry (LC–MS/MS) analysis was performed with ion-pairing reverse phase chromatography using an Ascentis Express column (C18, 5 cm × 2.1 mm, 2.7 mm, Sigma) and a Waters Xevo TQ-S triple quadrupole mass spectrometer. The LC solvents were 10 mM tributylamine and 15 mM acetic acid in 97:3 water:methanol (Solvent A), and methanol (Solvent B). LC and MS parameters were as previously reported^83,84^. In brief, elution from the column was performed over 12 min with the following gradient: *t* = 0, 0% solvent B, flow rate 0.4 ml min^−1^; *t* = 1, 0% solvent B, flow rate 0.4 ml min^−1^; *t* = 2, 20% solvent B, flow rate 0.3 ml min^−1^; *t* = 3, 20% solvent B, flow rate 0.25 ml min^−1^; *t* = 5, 55% solvent B, flow rate 0.15 ml min^−1^; *t* = 8, 95% solvent B, flow rate 0.15 ml min^−1^; *t* = 9.5, 95% solvent B, flow rate 0.15 ml min^−1^; *t* = 10, 0% solvent B, flow rate 0.4 ml min^−1^; and *t* = 12, 0% solvent B, flow rate 0.4 ml min^−1^. Mass spectra were acquired using negative-mode electrospray ionization operating in multiple-reaction monitoring (MRM) mode. The capillary voltage was 3,000 V and cone voltage was 50 V. Nitrogen was used as cone gas and desolvation gas, with flow rates of 150 and 600 l h^−1^, respectively. The source temperature was 150 °C and desolvation temperature was 500 °C. Argon was used as collision gas at a manifold pressure of 4.3 × 10^−3 ^mbar. Precursor and product ion *m*/*z*, collision energies and source cone potentials were optimized for each transition using Waters QuanOptimize software. RAW data folders were converted to mzXML using ProteoWizard^85^ and OpenMS^86^. Peak quantification was performed in MAVEN^87^ and data for each sample was normalized to PIPES peak intensity. For isotopic ratios, natural isotope abundance correction was performed using IsoCor^88^.

#### Lipidomics

##### Lipid extraction

A total of 700 μl of sample (4 μl of plasma diluted in water or 700 μl of homogenized cells) was mixed with 800 μl 1 N HCl:CH_3_OH 1:8 (v/v), 900 μl CHCl_3_, 200 μg ml^−1^ of the antioxidant 2,6-di-tert-butyl-4-methylphenol (BHT; Sigma-Aldrich) and 3 μl of SPLASH LIPIDOMIX Mass Spec Standard (Avanti Polar Lipids, 330,707). After vortexing and centrifugation, the lower organic fraction was collected and evaporated using a Savant Speedvac spd111v (Thermo Fisher Scientific) at room temperature and the remaining lipid pellet was stored at −20 °C under argon.

##### Mass spectrometry

Just before MS analysis, lipid pellets were reconstituted in 100% ethanol. Lipid species were analysed by LC electrospray ionization tandem mass spectrometry (LC–ESI/MS/MS) on a Nexera X2 UHPLC system (Shimadzu) coupled with hybrid triple quadrupole/linear ion trap mass spectrometer (6500+QTRAP system; AB SCIEX). Chromatographic separation was performed on a XBridge amide column (150 mm × 4.6 mm, 3.5 μm; Waters) maintained at 35 °C using mobile phase A (1 mM ammonium acetate in water-acetonitrile 5:95 (v/v)) and mobile phase B (1 mM ammonium acetate in water-acetonitrile 50:50 (v/v)) in the following gradient: (0–6 min: 0% B → 6% B; 6–10 min: 6% B → 25% B; 10–11 min: 25% B → 98% B; 11–13 min: 98% B → 100% B; 13–19 min: 100% B; and 19–24 min: 0% B) at a flow rate of 0.7 ml min^−1^, which was increased to 1.5 ml min^−1^ from 13 min onwards. SM, CE, CER, DCER, HCER and LCER were measured in positive ion mode with a precursor scan of 184.1, 369.4, 264.4, 266.4, 264.4 and 264.4 respectively. TAG, DAG and MAG were measured in positive ion mode with a neutral loss scan for one of the fatty acyl moieties. Phosphatidylcholine, lysohosphatidylcholine, phosphatidylethanolamine, lysophosphatidylethanolamine, phosphatidylglycerol, phosphatidylinositol and phosphatidylserine were measured in negative ion mode by fatty acyl fragment ions. Lipid quantification was performed by scheduled MRM, the transitions being based on the neutral losses or the typical product ions as described above. The instrument parameters were as follows: curtain gas = 35 psi; collision gas = 8 a.u. (medium); ionspray voltage = 5,500 V and −4,500 V; temperature = 550 °C; ion source gas 1 = 50 psi; ion source gas 2 = 60 psi; declustering potential = 60 V and −80 V; entrance potential = 10 V and −10 V; collision cell exit potential = 15 V and −15 V.

#### Membrane fluidity assay

OT-I T cells were isolated and cultured as described above. Membrane fluidity was assessed on day 7 of culture by using the Membrane Fluidity kit (Abcam) according to the manufacturer’s guidelines.

#### Cholesterol measurement

##### FILIPIN III staining

Cells were stained with a viability dye for 30 min at 4 °C, fixed with 4% paraformaldehyde for 30 min at 4 °C, and stained with 50 μg ml^−1^ FILIPIN III for 30 min at room temperature. Samples were then acquired with a FACS Fortessa X-20.

##### ALOD4 staining

ALOD4 protein was synthesized and conjugated as previously reported^41^. Then, 2 × 10^6^ cells per ml were stained in pure RPMI with 2 mg ml^−1^ with conjugated ALOD4 30 min at 37 °C as previously reported^39^. Then, staining with viability dye and antibodies was performed for 30 min at 4 °C. Samples were washed and freshly acquired with a FACS Fortessa X-20.

##### Biochemical measurement of total cholesterol levels

Cells were collected and lysed with complete RIPA buffer for 30 min at 4 °C. Total cellular cholesterol level was then quantified using the Amplex Red cholesterol assay kit (Invitrogen) according to the manufacturer’s guidelines.

#### In vitro memory assay

OT-I T cells were isolated and activated in presence of 10 ng ml^−1^ mIL-2. From day 4, cells were cultured in the presence of 5 ng ml^−1^ mIL-7 and 5 ng ml^−1^ mIL-15 to induce memory differentiation. On day 7, cells were collected and stained for FACS to assess the expression of CD62L and CD44 as a readout of memory differentiation.

#### Immunoblotting

TCR signalling was assessed by immunoblotting of proximal and downstream proteins of the TCR. In brief, naive CD8^+^ T cells were isolated and treated with 5 μM ELOVL1 inhibitor (C3) or DMSO for 6 h and then stimulated with 2 μg ml^−1^ soluble anti-CD3 and 5 μg ml^−1^ anti-CD28 for the indicated time and collected. For INSIG1 and mitochondrial complexes quantification, CD8^+^ T cells were isolated, activated and nucleofected as described above, and then collected on day 5 and day 7 from activation respectively. Immunoblotting on whole-cell lysate was performed as previously described^89^. The following antibodies were used: rabbit anti-LCK (1:2,000 dilution), rabbit anti-pLCK (1:2,000 dilution), rabbit anti-ZAP70 (1:2,000 dilution), rabbit anti-pZAP70 (1:2,000 dilution), rabbit anti-ERK1/2 (1:2,000 dilution), rabbit anti-pERK1/2 (1:2,000 dilution), rabbit anti-INSIG1 (1:1,000 dilution), mouse anti-OxPhos (1:1,000 dilution), anti-loading control HRP (1:2,000 dilution), mouse anti-vinculin (1:2,000 dilution), rabbit anti-β-actin (1:2,000 dilution) and appropriate HRP-conjugated secondary antibodies (1:3,000 dilution). The signal was visualized by enhanced chemiluminescent reagents (ECL, Invitrogen) or West Femto (Thermo Scientific), according to the manufacturer’s instructions, and images were acquired by a LAS-4000-CCD camera with ImageQuant software (GE Healthcare).

#### Peripheral blood mononuclear cell isolation and CD8^+^ T cell selection

Buffy coat samples from healthy donors were obtained from the Red Cross Donor Center Mechelen, Belgium (institutional approval S68611). Peripheral blood mononuclear cells were obtained by Ficoll density centrifugation (Axis-Shield, 1114545) and washed in PBS containing 1 mM EDTA. The ring at the interface was collected, washed with PBS and counted. CD8^+^ T cells were isolated using the human CD8^+^ T cells Isolation kit (MojoSort) according to the manufacturer’s guidelines. Isolated T cells were cultured for 24 h in T cell with 1:1 ratio of Human T-Activator CD3/CD28 Dynabeads (Thermo Fisher Scientific). For the following 2 days activated CD8^+^ T cells were expanded in T cell medium supplemented with 20 ng ml^−1^ hIL-2. For Elovl1 inhibition, hCD8^+^ T cells were treated for 3 days with 5 µM of ELOVL1 inhibitor (Medchem) and used for in vitro experiments.

#### Proliferation assays

##### Incucyte

CD8^+^ T cells were isolated from C57BL/6J mice or from the human buffy coat as described above. Then, 2 × 10^5^ CD8^+^ T cells were then seeded in T cell medium with a 1:1 ratio of Mouse or Human T-Activator CD3/CD28 Dynabeads (Thermo Fisher Scientific) on nontreated 48-well plates (Corning) coated with RetroNectin (Takara Bio) in the presence of 5 µM ELOVL1 inhibitor (MedChem) or DMSO as a control. Cell growth was monitored with an S3 Incucyte (Essen BioScience) for 100 h (optical module S3/SX1 G/R). Phase-contrast images were taken at 2-h intervals for the duration of the experiments. Cell proliferation was calculated by analysing the occupied area of cells with the Incucyte Base analysis software. Growth was calculated as maximal growth minus initial occupancy.

##### Cell trace violet dilution

CD8^+^ T cells were isolated from C57BL/6J mice and stained with cell trace violet (CTV). Upon a 6-h incubation with 5 µM ELOVL1 inhibitor (MedChem) or DMSO as a control in T cell medium, 5 × 10^4^ T cells were activated with a 1:1 ratio of Mouse or Human T-Activator CD3/CD28 Dynabeads (Thermo Fisher Scientific) in a 96-well plate (corning). Cells were collected 48 h later and analysed via FACS for CTV dilution and the absolute number was calculated using counting beads.

#### dSTORM-TIRF imaging

Naive CD8^+^ T cells were incubated for 6 h with DMSO or C3. Then, 2.5 × 10^5^ cells of each condition were plated on a polylysine-coated Nunc Lab-Tek Chambered Coverglass and incubated for 1 h at 37 °C to let them attach. Next, cells were fixed for 10 min at 4 °C with 4% paraformaldehyde, stained with 1 μg ml^−1^ soluble Alexa Fluor647 (AF647)-conjugated anti-CD3, 1 μg ml^−1^ and 5 μg ml^−1^ of unconjugated anti-CD3 and anti-CD28, respectively, for 10 min at 37 °C. Samples were washed twice and imaged.

Samples prepared in a Nunc Lab-Tek Chambered Coverglass (Cellvis, C8-1.5H-N) were placed in 1 ml dSTORM imaging buffer and the chamber was sealed with parafilm to prevent the entry of oxygen into the sample. dSTORM buffer consisted of 50 mM cysteamine hydrochloride (Sigma-Aldrich, M6500) and an oxygen scavenger system consisting of 10% d-glucose (Sigma-Aldrich, G7528), 0.5 mg ml^−1^ glucose oxidase from *Aspergillus* *niger* (Sigma-Aldrich, G7141) and 40 µg ml^−1^ catalase from bovine liver (Sigma-Aldrich, C40) in PBS 1× pH 7.4 as previously described^90^. dSTORM imaging was performed on a home-built microscope with built-in through-the-objective TIRF illumination. This IX83 inverted microscope (Olympus IX83 frame S1F-3, Olympus Optical) has been previously described^91^ and includes dichroic mirrors to combine the laser lines, neutral density filters to adjust the laser intensity (Newport Corporation), a 10× beam expander (Linos, Qioptiq), a focusing lens to enable TIRF illumination (f1/4 500 mm, BK 7, Newport Corporation), a ×60 TIRF oil objective (Olympus Optical, NA 1.49), a z488/561 dichroic mirror to separate excitation and emission, and a ×3.3 projection lens (Olympus) placed before a 512 × 512 pixels EM-CCD camera (ImagEM, Hamamatsu Photonics) to achieve a pixel size of approximately 80 nm.

For image acquisition, first, a diffraction-limited image was taken by 642 nm excitation (642 nm diode laser, Sapphire Coherent or Cobolt) that passed a series of neutral density filters (2.04 OD) before reaching the sample, while emission was captured above 655 nm (HQ 655 long-pass filter, Chroma Technology). For dSTORM imaging, AF647 was switched to the nonfluorescent intermediate state by continuous illumination with 642 nm light (neutral density filters 0.08 OD), and afterwards stochastically activated using 488 nm light (488 nm diode laser, Sapphire, Coherent, 1.54–2.54 OD). Fluorophore blinking was acquired by recording 1 × 10^4^ frames with an exposure time of 0.030530 s per frame. Cells were visualized in the transmission and diffraction-limited channel before starting the image acquisition to ensure that only attached single cells were imaged.

#### Mitochondria staining and confocal imaging

A total of 2 × 10^5^ sgNT or sgE*lovl1* OT-I T cells at day 7 after in vitro activation were seeded on a polylysine-coated 35-mm dish coverslip (Mattek, P35G-1.5-14-C) and incubated with MitoTracker Far Red (100 nM) for 30 min at 37 °C in Krebs solution (150 mM NaCl, 5.9 mM KCl, 1.2 mM MgCl_2_, 11.6 mM HEPES (pH 7.3), 11.5 mM glucose and 1.5 mM CaCl_2_), followed by Hoechst nuclear staining (10 µM) for 10 min at room temperature. Cells were washed twice in Krebs solution and high-resolution confocal images were taken as previously shown^92^.

### Quantification and statistical analysis

#### Data analysis of CRISPR screen

The multi-organ CRISPR/Cas9 screen of 2,078 genes involved in cellular metabolism was performed using a lentiviral library encoding 10,390 specific sgRNAs and 250 nontargeting control sgRNAs. MAGeCK-VISPR (v.0.5.3) was used to process CRISPR/Cas9 screen sequencing data. MAGeCK ‘count’ module generated raw count table with sgRNA as rows and samples as columns. This table was normalized by 250 nontargeting control sgRNAs and corrected for batches effects between different organs by Combat. Two samples were excluded after initial quality control. MAGeCK ‘mle’ module calculated *β* score for each targeted gene to measure positive or negative selection. STARS method was performed via http://pinapl-py.ucsd.edu/.

GO was performed via https://geneontology.org/docs/go-enrichment-analysis/.

#### Data analysis of scRNA-seq (CROP-seq)

CROP-seq was performed on a 10x platform. scRNA-seq generated data in two separate libraries: the Gene Expression library and CRISPR Guide Capture library. Raw reads (.fastq format) from the Gene Expression library were mapped to mouse genome (mm10) by 10x Genomics Cell Ranger (v.3.1.0). A Cell Ranger feature barcoding analysis pipeline was applied to processes reads from the CRISPR Guide Capture library. This pipeline searches reads against our designed sgRNA spacer sequences and returned sgRNA to cell assignment using an automatically determined unique molecular identifier (UMI) thresholds.

We took cells with single sgRNA detected to perform downstream analysis by the R package Seurat (v.3.2.3)^93^. We applied quality control to filter out cells with too high or low number of UMIs to eliminate possible doublets and dropouts. The cutoffs were determined in each sample separately. We also removed cells with more than 10% mitochondrial gene expression. For 22,371 cells after filtering, we performed normalization with default global-scaling-normalization methods, and then canonical correlation analysis integration to remove batch effect between two batches with default parameters. We scaled integrated data regressing out number of UMIs, mitochondrial counts per cent and the difference score between the G2M and S phase. In this way, we partially regressed out the cell-cycling effect while maintaining cell-differentiating processes as much as possible. We then performed principal-component analysis and Uniform Manifold Approximation and Projection (UMAP) on the first 15 principal components, which captured most of the variance. Cell clustering with resolution of 0.3 gave seven clusters, and biomarkers for each cluster were identified by the FindMarkers function. Cell type annotation was conducted based on marker genes.

#### Data analysis of bulk-RNA-seq

In vitro*,* read alignment was performed using the default parameters of STAR (v.2.7.10b) with the *Mus* *musculus* reference genome GRCm39. This alignment resulted in an average of 24.1 million uniquely mapped reads per sample. Aligned reads were quantified using featureCounts (v.2.0.1) in R (v.4.3.3) with options -t gene and -s 2. To reduce mice-to-mice variability, counts from replicate mice from the same experiment were merged before subsequent analyses. Differentially expressed genes were identified with the DESeq2 package (v.1.42.0) using the likelihood-ratio test test^94^. A list of all differentially expressed genes between control and *Elovl1* KO treated cells can be found in Supplementary Table 1. Data was visualized using ggplot2 (v.3.4.3) and stringr (v.1.5.0). GO analyses, using up- and downregulated differentially expressed genes (defined as genes with adjusted *P* < 0.05) as input, were performed using the enrichR package (v.3.2) using the ‘GO_Biological_Process_2023’ database^95^. A list of enriched terms can be found in Supplementary Table 1.

In vivo*,* raw sequencing reads were mapped to mm10 reference genome by STAR (v.2.7.7a) and RSEM^96^ (v.1.3.1) was used to quantify gene counts per sample. R package DESeq2 (ref. ^94^) (v.1.36.0) performed gene counts normalization and differential gene expression analysis. R package clusterProfiler^97^ (v.4.4.4) performed GSEA and ComplexHeatmap^98^ (v.2.12.1) made heatmap visualization. GO analyses were performed using up- and downregulated differentially expressed genes (defined as genes with adjusted *P* < 0.05 and −log_2_ fold change ± 1) as input. Revigo was used to group the redundant processes^99^.

#### Lipidomic data analysis

Peak integration was performed with the MultiQuant software (v.3.0.3). Lipid species signals were corrected for isotopic contributions (calculated with Python Molmass 2019.1.1) and were quantified based on internal standard signals and adheres to the guidelines of the Lipidomics Standards Initiative (level 2-type quantification).

#### Analysis of human scRNA-seq dataset

We collected scRNA-seq data of CD8^+^ T cells from a published melanoma dataset^35^. R package Survminer (v.0.4.9) and survival (v.3.5-7)^100^ were used to perform Kaplan–Meier analysis. The cutoff point of *ELOVL1* high and low expression was determined by the surv_cutpoint function.

#### dSTORM-TIRF analysis

dSTORM images were reconstructed using a custom-written MATLAB algorithm (MATLAB, R2022a) based on the open-source localizer software package^101^ that has previously been applied for SMLM analyses^102^. The centre position of the emitters in each of the 1 × 10^4^ frames was localized by fitting a two-dimensional Gaussian with PSF s.d. factor of 1.8 and intensity selection sigma factor of 25.

CD3 clustering was analysed by manually selecting a region of interest (containing the cell) from the imaged field of view and running a Voronoi analysis on the calculated molecular localizations (https://nl.mathworks.com/help/matlab/ref/voronoin.html). Voronoi areas corresponding to a molecular density three times higher than the average density were selected and adjacent selected Voronoi areas were grouped in clusters. Clusters with more than ten localizations were further analysed and the area and number of localizations were calculated for each cluster; as well as the area of the region of interest and the number of localizations included in it. Downstream analyses for calculation of the mean fraction of clustered localizations (mean of the number of localizations per cluster versus total number of localizations in all clusters, per region of interest), and localization density (total number of localizations in the region of interest versus area of the region of interest) were performed in Excel and statistical analyses were calculated in GraphPad Prism.

#### Mitochondria confocal analysis

Mitochondrial morphology was measured with Imaris (v.10.1) calculating the total mitochondrial volume and the index of mitochondrial fragmentation as previously shown^92^.

### Statistical analysis

Flow cytometry data were analysed using FlowJo v.10 on appropriate gated cells after removal of doublets and dead cells. Western blots were quantified with ImageJ software. All statistical analyses were performed using GraphPad Prism 10 software. In brief, comparisons for two groups were calculated using two-tailed Student’s *t*-tests. Comparisons of more than two groups were calculated using one-way analysis of variance (ANOVA) with Tukey multiple comparison correction. Data distribution was assumed to be normal but this was not formally tested. Detection of mathematical outliers was performed using the Grubbs’ test in GraphPad, and significant outliers were excluded. Results are represented as mean ± s.e.m. Statistical details are provided in figure legends, as well as sample size and number of independent repeats. No statistical methods were used to pre-determine sample sizes but our sample sizes are similar to those reported in previous publications^13,89^.

#### Randomization

For in vitro experiments, cells were randomly allocated to each treatment group. For in vivo, previous adoptive cell transfer, mice were randomized based on their body weight or tumour size to have similar average body weight or tumour size and s.d. in each group.

#### Blinding

For in vivo studies, the tumour measurement, treatment and analysis were performed without knowing the group code to ensure that the studies were run in a blinded manner. For in vitro studies, blinding of cell types was not possible.

### Reporting summary

Further information on research design is available in the Nature Portfolio Reporting Summary linked to this article.

## Supplementary information

Supplementary Information Supplementary gating strategy and legends.

Reporting Summary

Supplementary Table 1 Enriched targets in MAGeCK and STARS; list of target genes in tumour, lung and liver of mice treated with anti-PD-1 in the multi-organ screening; target genes ranked per cluster and combined rank from CROP-seq; CROP-seq library; sequences of primers and sgRNA for in vitro experiments; differentially expressed genes from vitro bulk-RNA-seq; differentially expressed genes from vivo bulk-RNA-seq.

## Source data

Source Data Fig. 1 Statistical source data.

Source Data Fig. 2 Statistical source data.

Source Data Fig. 3 Statistical source data.

Source Data Fig. 4 Statistical source data.

Source Data Fig. 5 Statistical source data.

Source Data Fig. 5 Unprocessed western blots.

Source Data Fig. 6 Statistical source data.

Source Data Fig. 6 Unprocessed western blots.

Source Data Fig. 7 Statistical source data.

Source Data Fig. 7 Unprocessed western blots.

Source Data Extended Data Fig. 1 Statistical source data.

Source Data Extended Data Fig. 2 Statistical source data.

Source Data Extended Data Fig. 3 Statistical source data.

Source Data Extended Data Fig. 4 Statistical source data.

Source Data Extended Data Fig. 5 Statistical source data.

Source Data Extended Data Fig. 6 Statistical source data.

Source Data Extended Data Fig. 7 Statistical source data.

## Data Availability

CRISPR NGS sequencing data, CROP-seq scRNA-seq data both unprocessed and processed reads, in vitro and in vivo bulk-RNA-seq data have been deposited at the Gene Expression Omnibus (GEO) and are publicly available with the following accession numbers: NGS (GSE255833); CROP-seq (GSE255832), in vitro bulk-RNA-seq (GSE282895) and in vivo bulk-RNA-seq (GSE282894). This paper analyses publicly available human melanoma and PDAC single-cell RNA-seq and human PDAC bulk-RNA-seq data from the original research article, deposited at GEO with the following accession numbers: Melanoma (GSE120575), PDAC scRNA-seq (GSE211644) and PDAC bulk (GSE179351). Source data are provided with this paper.

## Code availability

Matlab dSTORM analysis script has been uploaded on GitHub and is available through Zenodo at 10.5281/zenodo.14175266 (ref. ^103^).
