# Supplementary material for: A functional single-cell metabolic survey identifies Elovl1 as a target to enhance CD8+ T cell fitness in solid tumours
Source: Nat Metab. 2025 Mar 10;7(3):508–30. doi: 10.1038/s42255-025-01233-w (PMC11946891; doi:10.1038/s42255-025-01233-w)
Supplement: Supplementary file 1 — Supplementary gating strategy and legends. [file 42255_2025_1233_MOESM1_ESM.pdf]

nature metabolism

Supplementary information https://doi.org/10.1038/s42255-025-01233-w

A functional single-cell metabolic survey identifies *Elovl1* as a target to enhance CD8$^+$ T cell fitness in solid tumours

In the format provided by the authors and unedited

Supplementary Data Figure 1

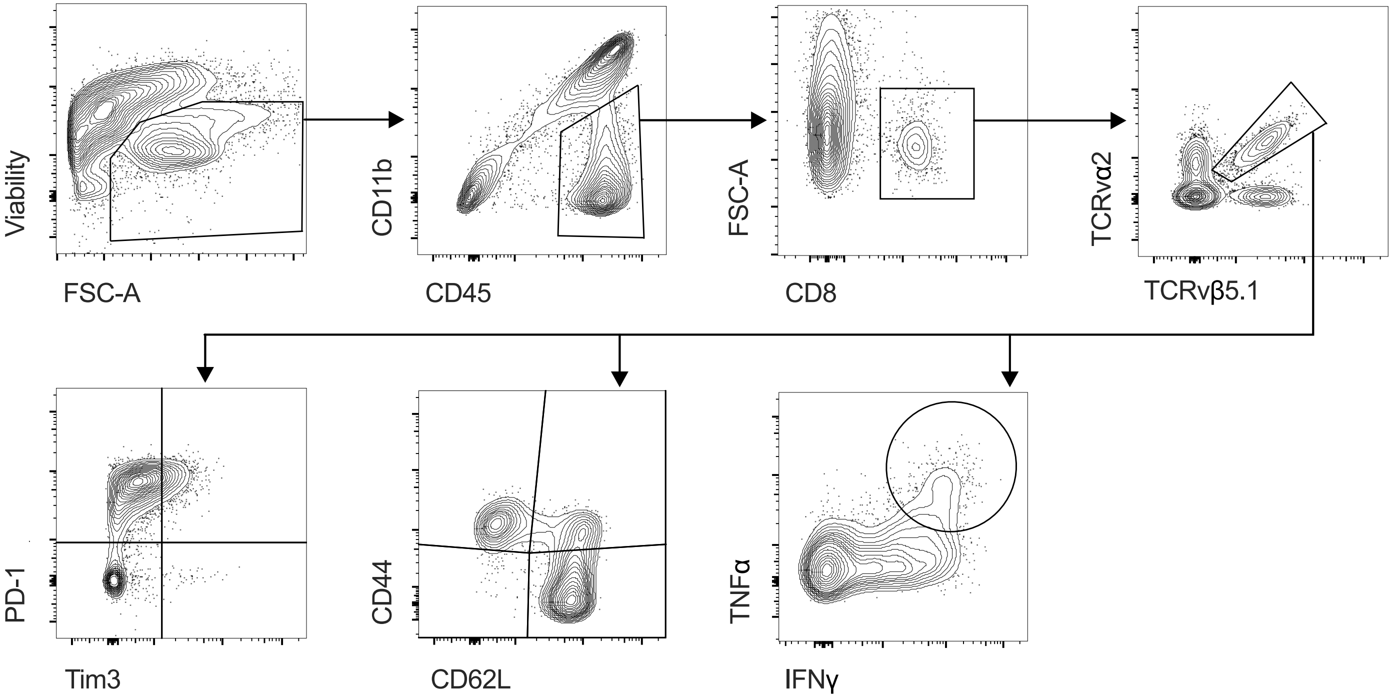

Representative gating strategy for *in vivo* OT-I T cells detection. OT-I T cells were identified using antibodies recognizing the OVA-specific alpha (TCRvα2) and beta chains (TCRvβ5.1). PD-1^+ Tim3^+ were gated out of OT-I T cells. T central memory (Tcm) cells were gated out of OT-I T cells as CD44^+ CD62L^+. Polyfunctional IFNγ^+ TNFα^+ cells were gated out of OT-I T cells.

This gating strategy was used for generating the data of main Figure 1, 2 and 3.

Supplementary Data Figure 2

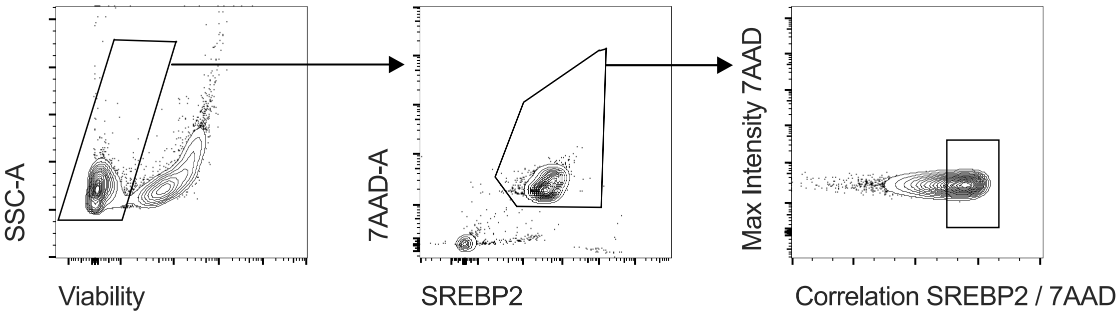

Representative gating strategy for SREBP2 nuclear translocation in *in vitro* cultured CD8$^+$ T cells performed with BD FACS Discovery S8 Cell Sorter. 7AAD was used to stain the cell nucleus of CD8$^+$ T cells. 7AAD$^+$ SREBP2$^+$ live CD8$^+$ T cells were gated to include only stained cells. The ratio SREBP2/7AAD was used as a redout for SREBP2 nuclear translocation.
This gating strategy was used for generating the data of main Figure 4i.
