# Supplementary material for: A functional single-cell metabolic survey identifies Elovl1 as a target to enhance CD8+ T cell fitness in solid tumours
Source: Nat Metab. 2025 Mar 10;7(3):508–30. doi: 10.1038/s42255-025-01233-w (PMC11946891; doi:10.1038/s42255-025-01233-w)
Supplement: Supplementary file 2 — Reporting Summary [file 42255_2025_1233_MOESM2_ESM.pdf]

nature portfolio

Corresponding author(s): Massimiliano Mazzone

Last updated by author(s): Jan 22, 2025

Reporting Summary

Nature Portfolio wishes to improve the reproducibility of the work that we publish. This form provides structure for consistency and transparency in reporting. For further information on Nature Portfolio policies, see our Editorial Policies and the Editorial Policy Checklist.

Statistics

For all statistical analyses, confirm that the following items are present in the figure legend, table legend, main text, or Methods section.

n/a	Confirmed	
☐	☒	The exact sample size (*n*) for each experimental group/condition, given as a discrete number and unit of measurement
☐	☒	A statement on whether measurements were taken from distinct samples or whether the same sample was measured repeatedly
☐	☒	The statistical test(s) used AND whether they are one- or two-sided<br>*Only common tests should be described solely by name; describe more complex techniques in the Methods section.*
☒	☐	A description of all covariates tested
☐	☒	A description of any assumptions or corrections, such as tests of normality and adjustment for multiple comparisons
☐	☒	A full description of the statistical parameters including central tendency (e.g. means) or other basic estimates (e.g. regression coefficient) AND variation (e.g. standard deviation) or associated estimates of uncertainty (e.g. confidence intervals)
☐	☒	For null hypothesis testing, the test statistic (e.g. *F*, *t*, *r*) with confidence intervals, effect sizes, degrees of freedom and *P* value noted<br>*Give P values as exact values whenever suitable.*
☒	☐	For Bayesian analysis, information on the choice of priors and Markov chain Monte Carlo settings
☒	☐	For hierarchical and complex designs, identification of the appropriate level for tests and full reporting of outcomes
☒	☐	Estimates of effect sizes (e.g. Cohen's *d*, Pearson's *r*), indicating how they were calculated

Our web collection on statistics for biologists contains articles on many of the points above.

Software and code

Policy information about availability of computer code

Data collection

QuantStudio TM 12K Flex software (v1.4) was used for real-time PCR. NGS sequencing was performed using an Illumina NextSeq 500 platform. Crop-seq sequencing was performed using an Illumina NovaSeq 6000 platform and reads were processed with the Cellranger (v3.1.0). In vitro bulk RNA sequencing was performed using Illumina Novaseq 6000 and reads were alligned with STAR (v2.7.10b). Aligned reads were quantified using featureCounts (v2.0.1) in R (v4.3.3). In vivo bulk RNA sequencing was performed using Illumina Novaseq X Plus and reads were alligned with STAR (v2.7.7a) and RSEM94 (v1.3.1) was used to quantify gene counts per sample. In vivo RNA ImageQuant software (v1.2) was use for immunoblot images acquisition. Incucyte Base analysis (v2018A) software was used for incucyte imaging collection. Flow cytometry data collection was done with BD FACs DIVA software (v9.0) and FACSChorus (v5.1) software. Seahorse was performed by seahorse XFe96 analyzer (Agilent). Lipidomics was performed via(LC-ESI/MS/MS) on a Nexera X2 UHPLC system (Shimadzu). For metabolomics samples were aquired with LC-MS/MS with ion-pairing reverse phase chromatography using an Ascentis Express column (C18, 5 cm x 2.1 mm, 2.7 mm, Sigma) and a Waters Xevo TQ-S triple quadrupole mass spectrometer. dSTORM images were aquired with a home-build microscope with built-in through-the-objective TIRF illumination. Mitochondrial were imaged on a high resolution confocal microscope.

Data analysis

All statistical analysis were performed using GraphPad Prism software (v10). NGS sequencing data were analyzed with MAGeCK-VISPR (v0.5.3) and STARS. CROP-seq sequencing data was analyzed with by R package Seurat (v3.2.3). R package Survminer (v0.4.9) and survival (v3.5-7) were used to perform Kaplan-Meier analysis from the Melanoma scRNA sequencing public dataset. In vitro bulk RNA seq was analysed using DESeq2 package (v1.42.0) and data were visualized with ggplot2 (v3.4.3) and stringr (v1.5.0). GO was performed using enrichR package (v3.2). In vivo bulk RNA seq was analysed using DESeq2 package (v1.36.0). GSEA was performed with clusterProfiler (v4.4.4) and data were visualized with ComplexHeatmap (v2.12.1). ImageJ (v1.53) was used for Western Blot image analysis. Incucyte Base analysis (v2018A) software was used for incucyte imaging analysis. Flow cytometry analysis was done with the FlowJo software (v10.8.1). OCR was analysed by seahorse wave software (seahorse, Agilent Technologies, v2.6). Lipidomics peak analysis was performed with MultiQuant™ software (3.0.3). Tracing

metabolomics analysis RAW data folders were converted to mzXML using ProteoWizard (v2.0) and OpenMS (3.1.0). Peak quantification was performed in MAVEN. Mitochondrial morphology was measured with Imaris (v10.1).

For manuscripts utilizing custom algorithms or software that are central to the research but not yet described in published literature, software must be made available to editors and reviewers. We strongly encourage code deposition in a community repository (e.g. GitHub). See the Nature Portfolio guidelines for submitting code & software for further information.

Data

Policy information about availability of data

All manuscripts must include a data availability statement. This statement should provide the following information, where applicable:

- Accession codes, unique identifiers, or web links for publicly available datasets
- A description of any restrictions on data availability
- For clinical datasets or third party data, please ensure that the statement adheres to our policy

CRISPR NGS sequencing data, CROP-seq single-cell RNA-seq data both unprocessed and processed reads, in vitro and in vivo bulk RNA-seq data have been deposited at GEO and are publicly available with the following accession numbers: NGS (GSE255833); CROP-seq (GSE255832), in vitro bulk RNA-seq (GSE282895), in vivo bulk RNA-seq (GSE282894). This paper analyses publicly available human Melanoma and PDAC single-cell RNA-seq and human PDAC bulk RNA-seq data from the original research article, deposited at GEO with the following accession numbers: Melanoma (GSE120575), PDAC scRNA seq (GSE211644), PDAC bulk (GSE179351).

Research involving human participants, their data, or biological material

Policy information about studies with human participants or human data. See also policy information about sex, gender (identity/presentation), and sexual orientation and race, ethnicity and racism.

Reporting on sex and gender	CD8+ T cells were isolated from buffy coats of healthy male and female volunteers aged between 25 and 65 years (anonymized).
Reporting on race, ethnicity, or other socially relevant groupings	No socially relevant categorization variable or people classification into categories was applied in this study.
Population characteristics	Buffy coats was collected from healthy male and female volunteers aged between 25 and 65 years.
Recruitment	Donors were healthy volunteers.
Ethics oversight	The study was approved by the Ethical Committee of the University Hospitals KU Leuven (Leuven, Belgium) with the reference number S68611. Donors provided written consent.

Note that full information on the approval of the study protocol must also be provided in the manuscript.

Field-specific reporting

Please select the one below that is the best fit for your research. If you are not sure, read the appropriate sections before making your selection.

☒ Life sciences ☐ Behavioural & social sciences ☐ Ecological, evolutionary & environmental sciences

For a reference copy of the document with all sections, see nature.com/documents/nr-reporting-summary-flat.pdf

Life sciences study design

All studies must disclose on these points even when the disclosure is negative.

Sample size	No statistical method was used to predetermine sample size, but our sample sizes were selected based on those reported in previous studies (Virga et al. Sci. Adv. 2021; Celus et al. Cancer Immunol. Res. 2022; Cappellesso et. al. Nat. Cancer 2023)
Data exclusions	Detection of mathematical outliers was performed using the Grubbs' test in GraphPad
Replication	For in vitro experiments at least two-three biological replicates (cell isolated from different individual mice) were performed with similar results. For in vivo studies at least 5 animals were allocated per group. When representative data are shown, the experimental findings were reproduced independently with similar results.
Randomization	For in vitro experiments cells were randomly allocated to each treatment group. For in vivo, prior adoptive cell transfer, mice were randomized based on their body weight or tumor size to have similar average body weight or tumor size and standard deviation in each group.
Blinding	For in vivo studies, the tumor measurement, treatment and analysis were performed blindly without knowing the group code to ensure that the studies were run in a blinded manner. For in vitro studies, blinding of cell types was not possible. However, cells were treated in the same way and most of these experiments had a machine-based readout, which is not subjected to investigator bias.

Reporting for specific materials, systems and methods

We require information from authors about some types of materials, experimental systems and methods used in many studies. Here, indicate whether each material, system or method listed is relevant to your study. If you are not sure if a list item applies to your research, read the appropriate section before selecting a response.

Materials & experimental systems

n/a	Involved in the study
☐	☒ Antibodies
☐	☒ Eukaryotic cell lines
☒	☐ Palaeontology and archaeology
☐	☒ Animals and other organisms
☒	☐ Clinical data
☒	☐ Dual use research of concern
☒	☐ Plants

Methods

n/a	Involved in the study
☒	☐ ChIP-seq
☐	☒ Flow cytometry
☒	☐ MRI-based neuroimaging

Antibodies

Antibodies used

For western blot:
INSIG-1 (Proteintech, Cat#55282-1P, polyclonal) 1/1000,
LCK (Cell signaling, Cat#2752, polyclonal) 1/2000,
pLCK (Cell signaling, Cat#2751, polyclonal) 1/2000,
ZAP70 (Cell signaling, Cat#3165, [D1C10E]) 1/2000,
pZAP70 (Cell signaling, Cat#2717, [65E4]) 1/2000,
ERK1/2 (Cell signaling, Cat#9102, polyclonal) 1/2000,
pERK1/2 (Cell signaling, Cat#9101, polyclonal) 1/2000,
OxPhos Human WB antibody (Thermo, Car#45-8199, coktail) 1/1000,
beta Actin (Abcam, Cat#21185, polyclonal) 1/2000,
vinculin (Sigma-Aldrich, Cat#V9131, polyclonal) 1/2000,
HRP-conjugated secondary antibodies (Cell Signalling, anti-mouse; 7076S, anti-rabbit; 7074S) 1/2000.

For flow cytometry:
CD8a (PE-Cy7, Alexa Fluor 488, Thermo Fisher Scientific, Cat#25-0081-82, Cat#53-0081-82, [53-6.7]) 1/200,
CD11b (eFluor 450, Thermo Fisher Scientific, Cat#48-0112-82, [M1/70]) 1/400,
Thy1.1 (CD90.1) (PE, Alexa Fluor 488, BioLegend, Cat#202524, Cat#202506, [OX-7]) 1/700,
Vα2 (PerCP/Cy5.5, BioLegend, Cat#127814, [B20.1]) 1/200,
TCRVb 5.1/5.2 (APC, PE, Thermo Fisher Scientific, Cat#17-5796-82, Cat#17-5796-82, [MR9-4]) 1/200,
CD45 (APC/Cy7, BUV395, BioLegend, Cat#103116, Cat#564279, [30-F11]) 1/400,
CD279 (PD-1) (PE, BV711, BioLegend, Cat#135206, Cat#13523, [29F.1A12]) 1/800,
CD366 (TIM3) (PE-Cy7, Thermo Fisher Scientific, Cat#25-5870-82, [RMT3-23]) 1/200,
Ly-108 (Slamf6) (Alexa Fluor 647, BD Biosciences, Cat#561547, [13G3])1/200,
CD39 (Pe/Dazzle594, Biolegend, 143812, [Duha59])1/400,
TIGIT (BV421, Biolegend, 142111, [1G8])1/200,
CD62L (L-Selectin) (APC Thermo, Fisher Scientific, Cat#17-0621-82, [MEL-14])1/200,
CD62L (L-Selectin) (BV650, BioLegend, Cat#104453, [MEL-14])1/200,
CD44 (BV510, BioLegend, Cat#103044, [IM7])1/200,
Granzyme B (Alexa Fluor 647, BioLegend, Cat#515406, [GB11])1/100,
IFN gamma (PE-Cy7, Thermo Fisher Scientific, Cat#25-7311-82, [XMG1.2])1/200,
IL-2 (APC/Fire 750, BioLegend, Cat#503842,[JES6-5H4])1/100,
TNF-α (BV421, BioLegend, Cat#506327, [MP6-XT22])1/400,
Ki-67 (Alexa Fluor 700, Thermo Fisher Scientific, Cat#56-5698-82, [SolA15])1/100,
CD107a (PE, Biolegend, [1D4B] 1/200 during the restimulation,
CD16/CD32 (BD Biosciences, Cat# 553142, [2.4G2]) 1/25,
LDLR (R&D Systems, Cat#AF2255) 1/100,
SREBP2 (Abcam, Cat# ab30682) 1/200,
Donkey anti-Goat secondary antibody (Alexa Fluor 647, Thermo Fisher Scientific, Cat#A-21447) 1/400,
Donkey anti-Rabbit secondary antibody (Alexa Fluor 488, Thermo Fisher Scientific, Cat# A-21206) 1/400,
Fixable Viability Dyes (Thermo Fisher Scientific; eFluor 450 1/500, Cat#65-0863-18; eFluor 506, Cat#65-0866-18 1/500; eFluor 780 Cat#65-0865-18, 1/600),
7-AAD (Biolegend, Cat#420404) 1/20.

For culture:
Anti-mouse CD28 unconjugated (BD Biosciences, Cat# 553295),
Ultra-LEAF™ Purified anti-mouse CD3 (BioLegend Cat#100360).

For in vivo experiments:
Rat serum IgG (Sigma-Aldrich, I4131) 100mg/ treatment/ mouse;
Ultra-LEAF™ Purified PD-1anti-mouse (CD279) (BioLegend, 96167, [RMP1-14]) 100mg/ treatment/ mouse.

	For dSTORM:<br>CD3e (A647, Biolegend, 100322, [145-2C11]) 1µg/ mL
Validation	The following commercially available antibodies were validated by the manufacturer company, as well as other researchers (as the information collected by the RRID database):<br>For western blot: LCK (Cell signaling, Cat#2752, polyclonal, RRID:AB_2234649), suitable for WB. Reacts with Human, mouse.<br>pLCK (Cell signaling, Cat#2751, polyclonal, RRID:AB_330446), , suitable for WB. Reacts with Human, mouse.<br>ZAP70 (Cell signaling, Cat#3165, [D1C10E], RRID:AB_2218656), suitable for WB. Reacts with Human, mouse.<br>pZAP70 (Cell signaling, Cat#2717, [65E4], RRID:AB_2218658), suitable for WB. Reacts with Human, mouse.<br>ERK1/2 (Cell signaling, Cat#9102, polyclonal, RRID:AB_330744), suitable for WB. Reacts with Human, Mouse, Rat, Hamster, Monkey, Mink, Zebrafish, Bovine, Pig, S. cerevisiae.<br>pERK1/2 (Cell signaling, Cat#9101, polyclonal, RRID:AB_331646), suitable for WB. Reacts with Human, Mouse, Rat, Hamster, Monkey, Mink, Zebrafish, Bovine, Pig, C. elegans.<br>Vinculin (Sigma-Aldrich, V9131, hVIN-1, RRID:AB_477629), suitable for WB. Reacts with bovine, canine, mouse, rat, turkey, human, chicken, frog.<br>For FACS analysis: CD8a (PE-Cy7, Alexa Fluor 488, Cat#25-0081-82, Cat#53-0081-82, RRID:AB_469584,RRID:AB_469897) , CD11b (eFluor 450, Cat#48-0112-82, RRID:AB_1582236), Thy1.1 (CD90.1) (PE, Alexa Fluor 488, Cat#202524, Cat#202506, RRID:AB_1595524, RRID:AB_492882), Vα2 (PerCP/Cy5.5, Cat#127814, RRID:AB_1186116), TCRVb 5.1/5.2 (APC, PE, Cat#17-5796-82, Cat#17-5796-82, RRID:AB_2573222, RRID:AB_2573222), CD45 (APC/Cy7, BUV395, Cat#103116, Cat#564279, RRID:AB_312981, RRID:AB_2651134), CD279 (PD-1) (PE, BV711, Cat#135206, Cat#13523, RRID:AB_1877231, RRID:AB_2566158), CD366 (TIM3) (PE-Cy7, Cat#25-5870-82, RRID:AB_2573483), Ly-108 (Slamf6) (Alexa Fluor 647, Cat#561547, RRID:AB_10712759), CD62L (L-Selectin) (APC Thermo, Cat#17-0621-82, RRID:AB_469410), CD62L (L-Selectin) (BV650, Cat#104453, RRID:AB_2800559), CD44 (BV510, Cat#103044, RRID:AB_2650923), Granzyme B (Alexa Fluor 647, Cat#515406, RRID:AB_2566333), IFN gamma (PE-Cy7, Cat#25-7311-82, RRID:AB_469680), IL-2 (APC/Fire 750, Cat#503842, RRID:AB_2832800), TNF-α (BV421, Cat#506327, RRID:AB_10900823), Ki-67 (Alexa Fluor 700, Cat#56-5698-82, RRID:AB_2637480), CD16/CD32 (Cat# 553142, RRID:AB_394657), LDLR (Cat#AF2255, RRID:AB_355203), SREBP2 (Cat# ab30682, RRID:AB_779079), Donkey anti-Goat secondary antibody (Alexa Fluor 647, Cat#A-21447, RRID:AB_2535864), Donkey anti-Rabbit secondary antibody (Alexa Fluor 488, Cat# A-21206, RRID:AB_2535792).<br>Additionally, FMO (fluorescence minus one) was evaluated for every antibody to assess specificity and gating in FACS stainings.

Eukaryotic cell lines

Policy information about cell lines and Sex and Gender in Research

Cell line source(s)	HEK-293T and B16F1 were purchased from ATCC. KPC 1245 (FC1245) murine pancreatic ductal adenocarcinoma cell line was kindly provided by Tuveson's lab at the Johns Hopkins University and it was generated from C57BL6 mice carrying different genetic mutations P48Cre/KrasG12D/p53LSL R172H. KPC 1245_OVA was generated in our lab by lentiviral transduction. Primary OT-I and CD8+ T cells were isolated from both male and female mice. Human CD8+ T were isolated from buffy coats of both female and males healthy donors provided by Red Cross Donor Center Mechelen (Belgium).
Authentication	Cell lines were authenticated based on morphological criteria. Once thawed, cells were not kept for longer than 10 passages in a humidified incubator in 5% CO2 and 95% air at 37 ºC. An internal golden stock of all cell lines was generated and maintained by the Lab Manager.
Mycoplasma contamination	All cell lines were confirmed to be mycoplasma-free by PlasmoTest™ - Mycoplasma Detection Kit (InvivoGen).
Commonly misidentified lines<br>(See ICLAC register)	No commonly misidentified cell lines were used.

Animals and other research organisms

Policy information about studies involving animals; ARRIVE guidelines recommended for reporting animal research, and Sex and Gender in Research

Laboratory animals	C57BL6/J, Rag2/OT-I and Pmel-1 mice were purchased from Taconic. Rosa26-Cas9 knockin immunocompetent mice, which constitutively express the Cas9 nuclease, were kindly provided by Dr. Jan Cools from VIB-KU Leuven. OT-I:Rosa26-Cas9 mice were generated by intercrossing Rag2/OT-I mice with Rosa26-Cas9 mice. All mice used for tumor experiments were female or male between 6 and 12 weeks old. Mice were maintained under pathogen-free, temperature- and humidity-controlled conditions under a 12/12-h light/dark cycle and received normal chow (ssniff® R/M-H).
Wild animals	No wild animals were used for this study.
Reporting on sex	The phenotypes were observed indiscriminately in male and female mice. No gender related issues are applied to this work.
Field-collected samples	No field-collected samples were used for this study
Ethics oversight	Housing and all experimental animal procedures were approved by the Institutional Animal Care and Research Advisory Committee of the KU Leuven (P226/2017) and the Landesverwaltungsamt, SA, and LANUV (81-02.04.2020.A355), NRW, Germany. Animals were removed from the study and killed if any signs of pain and distress were detected, if they lost more than 20% of body weight or if the tumor volume reached 1500mm3. The maximal tumor size was not exceeded in all reported studies.

Note that full information on the approval of the study protocol must also be provided in the manuscript.

Plants

Seed stocks

Report on the source of all seed stocks or other plant material used. If applicable, state the seed stock centre and catalogue number. If plant specimens were collected from the field, describe the collection location, date and sampling procedures.

Novel plant genotypes

Describe the methods by which all novel plant genotypes were produced. This includes those generated by transgenic approaches, gene editing, chemical/radiation-based mutagenesis and hybridization. For transgenic lines, describe the transformation method, the number of independent lines analyzed and the generation upon which experiments were performed. For gene-edited lines, describe the editor used, the endogenous sequence targeted for editing, the targeting guide RNA sequence (if applicable) and how the editor was applied.

Authentication

Describe any authentication procedures for each seed stock used or novel genotype generated. Describe any experiments used to assess the effect of a mutation and, where applicable, how potential secondary effects (e.g. second site T-DNA insertions, mosiacism, off-target gene editing) were examined.

Flow Cytometry

Plots

Confirm that:

☒ The axis labels state the marker and fluorochrome used (e.g. CD4-FITC).

☒ The axis scales are clearly visible. Include numbers along axes only for bottom left plot of group (a 'group' is an analysis of identical markers).

☒ All plots are contour plots with outliers or pseudocolor plots.

☒ A numerical value for number of cells or percentage (with statistics) is provided.

Methodology

Sample preparation

Mice were sacrificed by cervical dislocation and the tumors were harvested in cold PBS. Tumors were minced in alpha MEM (Lonza) containing 0,085 mg/ml Collagenase V (Sigma), 0,125 mg/ml Collagenase D (Roche), 0,1 mg/ml Dispase (Gibco), 5U/ml DNase I (Sigma) and 50 µM mercaptoethanol (Gibco). Tumor pieces were collected into gentleMACS C tubes (Miltenyi Biotec) and dissociated by using first the h_cord_1 program of an automatic tissue gentleMACS Dissociator (Miltenyi Biotec) and then incubated for 40 minutes at 37°C.
Peritoneal metastases were collected and mechanically dissociated in 5 ml of the same digestion buffer used for the primary tumor. The pieces were then collected into gentleMACS C tubes (Miltenyi Biotec) and dissociated by using the 37C_m_TDK_1 program.
Lungs and livers were collected and dissociated with 10 ml of lung and liver Digestion Buffer (RPMI supplemented with 1% Pen/Strep, 5% FBS, 40U/mL DNase I (Sigma-Aldrich), 1 mg/mL Collagenase I (Sigma-Aldrich) and 2 mg/mL Dispase (Gibco) in C tubes (Miltenyi Biotec) using the 37C_m_LDK_1 program.
The digested tissues were filtered using a 70-µm pore sized strainer and cells were centrifuged 5 minutes at 300 xg.
Spleen and lymph nodes were mechanically dissociated in a 70-µm pore sized strainer and cell were centrifuged 5 minutes at 300 xg.
Red blood cell lysis was performed by using Red Blood Cell Lysing Buffer (Sigma-Aldrich), incubated for 2 minutes at 37°C, washed, and filtered through a 40-µm pore sized strainer.

Instrument

FACS LRSFortessa X-20 (BD Bioscience, model number 658226R1). FACS Aria III (BD Bioscience, model number 648282), FACSDiscover S8 Cell Sorter (BD Bioscience)

Software

Flow cytometry data collection was done with BD FACs DIVA software (v9.0) and FACSChorus software, for cell analysis and SREBP2 nuclear translocation respectively.
Flow cytometry analysis was done with the FlowJo software (v10.8.1).

Cell population abundance

Cell sorting was used for the screenings to sort OT-I T cells population for subsequent NGS or single-cell RNA sequencing.
In the case of NGS screening, genomic DNA was isolated from sorted OT-I T cells and sgRNA region integrated in the genomic DNA was amplified via PCR. Therefore, eventual non transduced OT-I T cells present in the sorted pool, were excluded via PCR. For CROP-seq, single cell suspension of CD90.1 positive OT-I T cells, were loaded into 10x genomics platform, and purity was assessed upon sequencing. Only single-cell Harboring one sgRNA sequence were used for following analysis.

Gating strategy

OT-I T cells were gated for FSC/SSC, CD45 positive/alive, CD11b negative, CD8 positive, TCRva2 and TCRvb5 double positive.
IFNgamma, TNF alpha double positive cells were gated out of OT-I T cells.
PD-1, TIM3 double positive cells were gated out of OT-I T cells.
T central memory cells (Tcm) we gated out of OT-I or Pmel-1 T cells as CD62L CD44 double positive.
For SREBP2 nuclear translocation T cells were gated as single and alive. Then 7AAD positive T cells (with nucleus stained) were gated and a correlation SREBP2/7AAD was used to determine the percentage of cells with SREBP2 translocated to the nucleus.

☒ Tick this box to confirm that a figure exemplifying the gating strategy is provided in the Supplementary Information.
