# Supplementary figures and images for: A functional single-cell metabolic survey identifies Elovl1 as a target to enhance CD8+ T cell fitness in solid tumours
Source: Nat Metab. 2025 Mar 10;7(3):508–30. doi: 10.1038/s42255-025-01233-w (PMC11946891; doi:10.1038/s42255-025-01233-w)

INSIG1 protein quantification  
Figure 5b

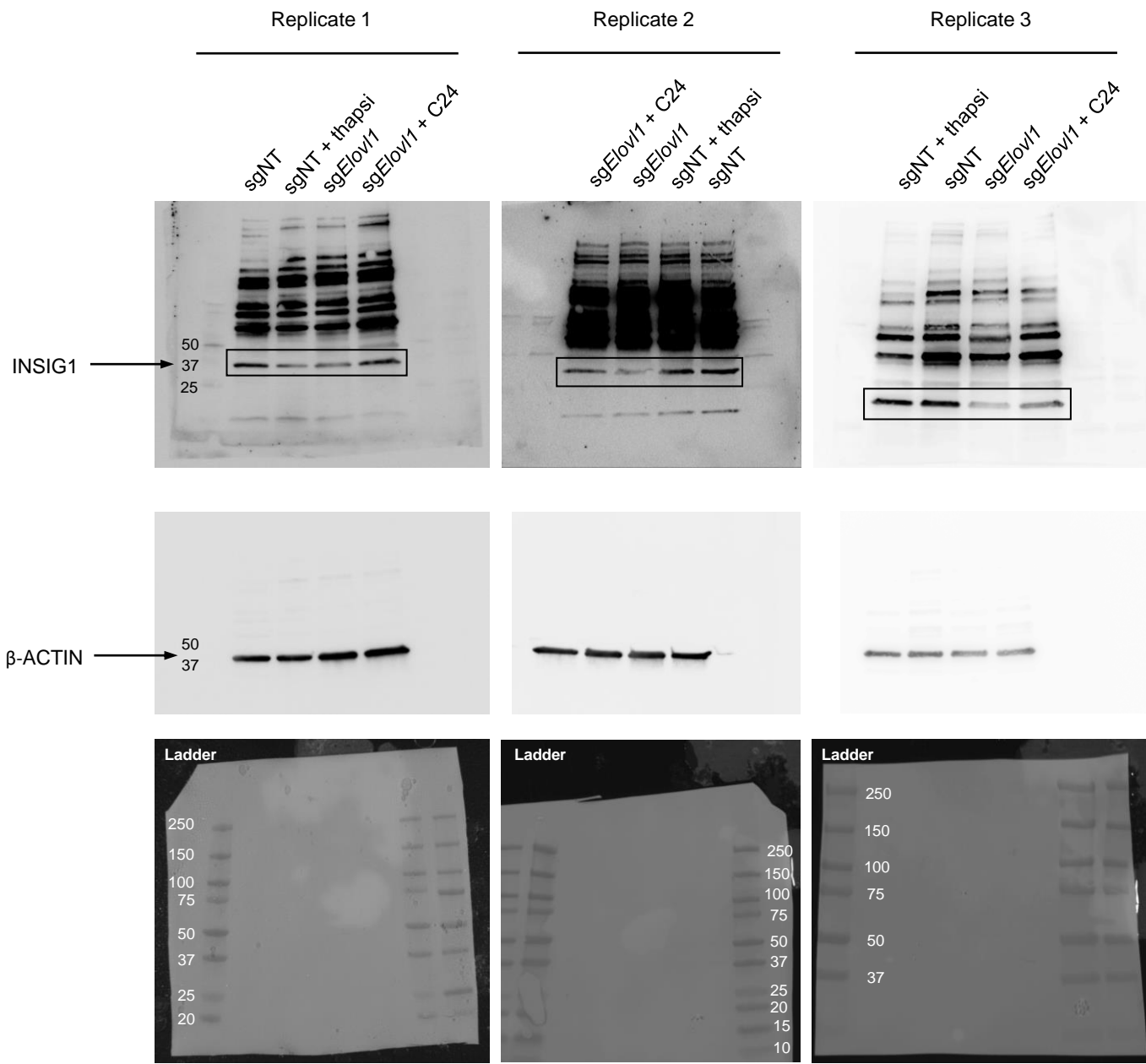

Supplement: Supplementary file 9 — Unprocessed western blots. [file 42255_2025_1233_MOESM9_ESM.pdf]

# TCR signaling on CD8<sup>+</sup> T cells pre-treated with ELOVL1 inhibitor or DMSO Figure 6g

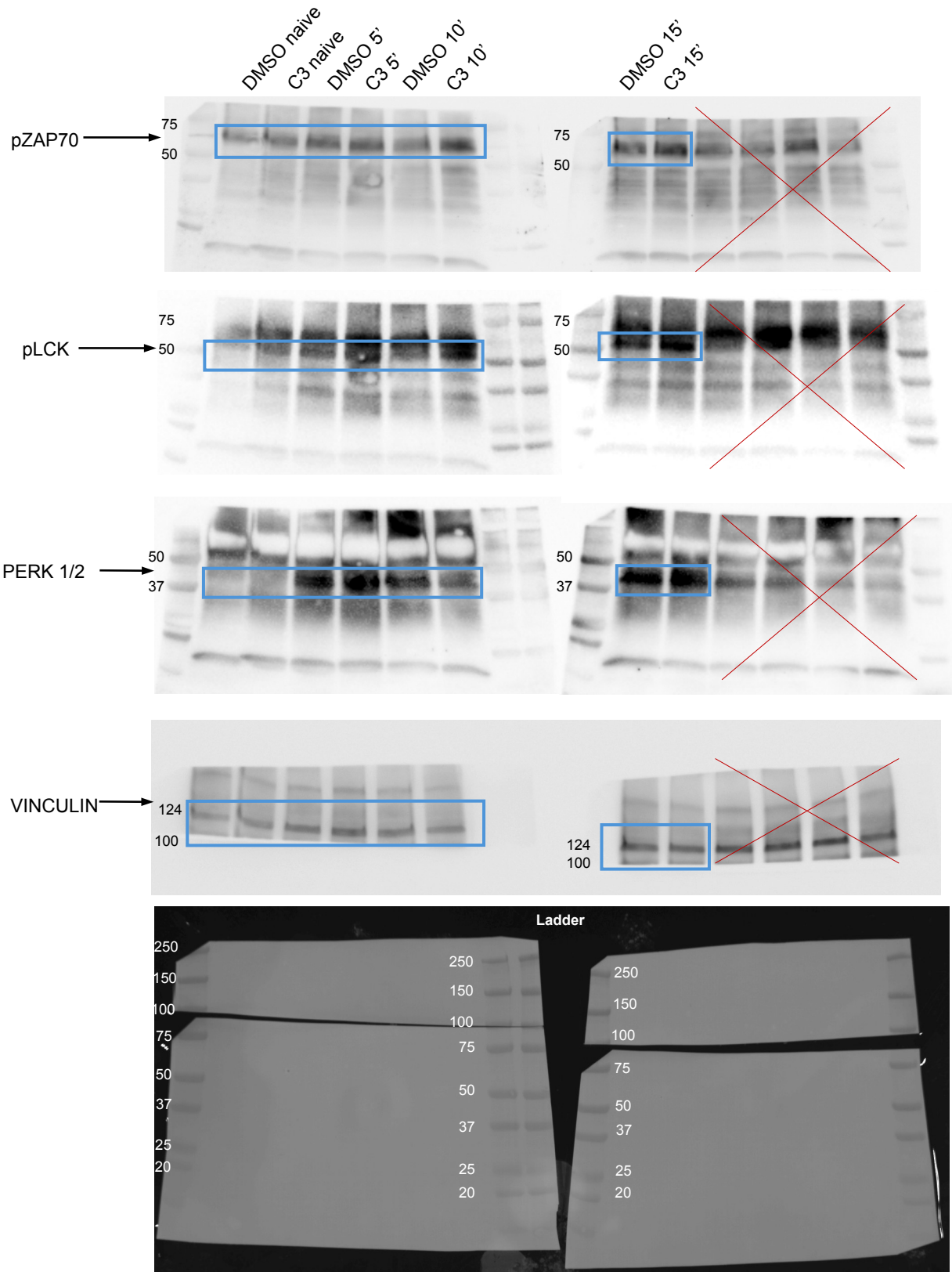

Supplement: Supplementary file 11 — Unprocessed western blots. [file 42255_2025_1233_MOESM11_ESM.pdf]

Electron transport chain protein quantification  
Figure 7d

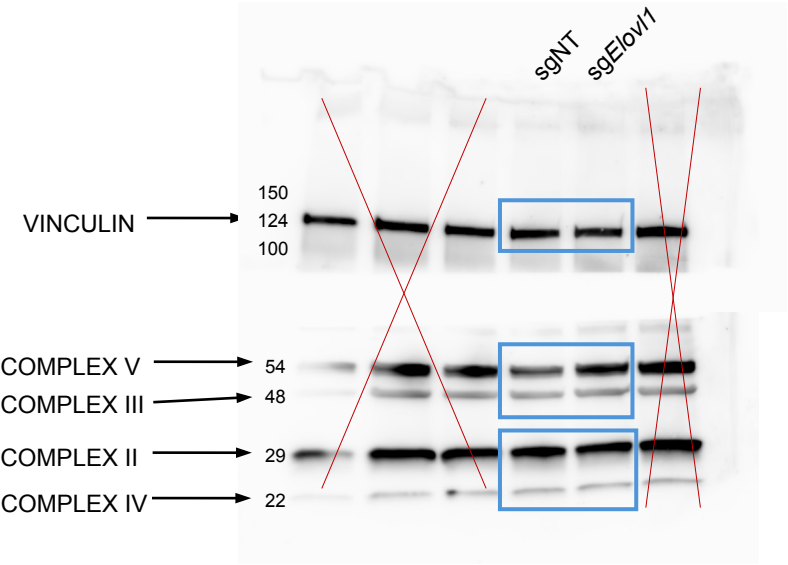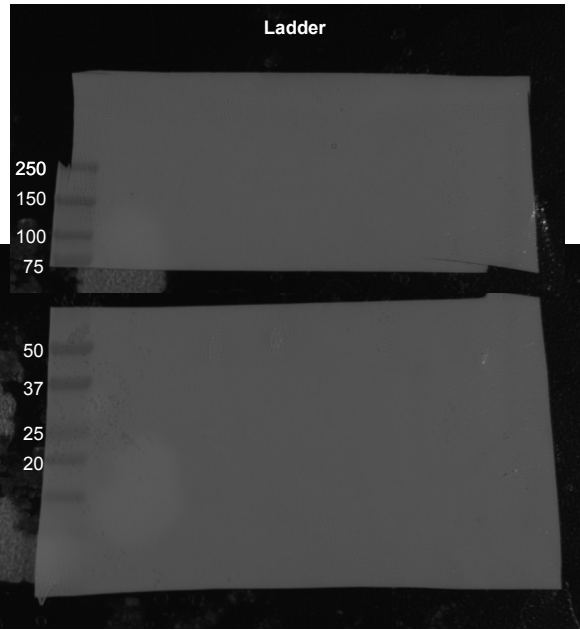

Supplement: Supplementary file 13 — Unprocessed western blots. [file 42255_2025_1233_MOESM13_ESM.pdf]
